# Provision of family planning vouchers and early initiation of postpartum contraceptive use among women living with HIV in southwestern Uganda: A randomized controlled trial

**DOI:** 10.1371/journal.pmed.1002832

**Published:** 2019-06-21

**Authors:** Esther C. Atukunda, Godfrey R. Mugyenyi, Celestino Obua, Elly B. Atuhumuza, Edward J. Lukyamuzi, Angela Kaida, Amon G. Agaba, Lynn T. Matthews

**Affiliations:** 1 Mbarara University of Science and Technology, Mbarara, Uganda; 2 Faculty of Health Sciences, Simon Fraser University, Burnaby, Vancouver, Canada; 3 Division of Infectious Diseases and Center for Global Health, Massachusetts General Hospital, Boston, Massachusetts, United States of America; 4 Division of Infectious Disease, University of Alabama at Birmingham, Birmingham, Alabama, United States of America; FHI360, UNITED STATES

## Abstract

**Background:**

Unwanted pregnancies remain a burden for women living with HIV (WLWH). Family planning prevents unplanned pregnancies while promoting longer birth intervals, key strategies to eliminate perinatal transmission of HIV and promote maternal and child health. We evaluated the effect of a family planning voucher, inclusive of immediate postpartum counseling, on uptake, early initiation, and continuation of modern contraceptive methods among recently postpartum WLWH delivering at a publicly funded regional referral hospital in rural, southwestern Uganda.

**Methods and findings:**

We performed a randomized controlled trial between October, 2016 and June, 2018 at a referral hospital in southwestern Uganda. This interim analysis includes adult WLWH randomized and enrolled equally to receive a family planning voucher or standard of care (control). Enrolled postpartum WLWH completed an interviewer-administered questionnaire at enrollment and 6 months postpartum. Our primary outcome of interest for this analysis is initiation of a modern family planning method within 8 weeks postpartum. Secondary outcomes included family planning initiation at 12, 14, 16, and 20 weeks postpartum, family planning discontinuation and/or change, pregnancy incidence, and mean time without contraception. The trial was registered with clinicaltrials.gov (NCT02964169). At enrollment, half of the women in both the voucher (N = 87, 55%) and control (N = 86, 54%) groups wanted to have a child in 2 years postpartum. Over 80% of referent pregnancies in the voucher (N = 136, 86%) and control (N = 128, 81%) groups were planned. All women were accessing ART. The mean CD4 count was 396 cells/mm^3^ (SD = 61) for those enrolled in the control group versus 393 cells/mm^3^ (SD = 64) in the family planning voucher group. By 8 weeks postpartum, family planning was initiated in 144 (91%) participants in the voucher group and 83 (52%) participants in the control group (odds ratio [OR] 9.42; CI 4.67–13.97, *P* < 0.001). We also found high family planning uptake rates for both groups, with higher rates among the intervention group at 12 weeks (OR 5.66; CI 2.65–12.12, *P* < 0.001), 14 weeks (OR 2.51; CI 1.31–4.79, *P* < 0.001), 16 weeks (OR 4.02; CI 1.66–9.77, *P* = 0.001), and 20 weeks (OR 3.65; CI 1.40–9.47, *P* = 0.004) postpartum. The average time to family planning initiation was reduced to 5.9 weeks (SD = 2.4) for those in the voucher group compared to 9.3 weeks (SD = 5) in the control (*P* < 0.001). One pregnancy was recorded in the group receiving standard of care; none were reported in the voucher group. Method mix did not differ by group: injectables were selected by most women (N = 150, 50%), and 52% of this proportion were in the experimental arm, with <10% in each arm selecting condoms, oral contraception, or intrauterine devices (IUDs). Similar proportions of women changed contraceptive methods over the 6-month follow-up in the voucher and control groups (N = 8, 5% versus N = 5, 4%; *P* = 0.467). More women in the control group discontinued contraception for 1 to 2 weeks (N = 19, 13% versus N = 7, 5%; *P* = 0.008) or more than 4 weeks (N = 15, 10% versus N = 3, 2%; *P* = 0.002) compared to those given a family planning voucher. The main limitation of this study is that its findings may not be generalized to settings without improved availability of contraceptives in publicly funded facilities.

**Conclusion:**

These findings indicate that a well-structured, time-bound family planning voucher program appeared to increase early postpartum contraceptive uptake and continuation in a setting in which users are faced with financial, knowledge, and structural barriers to contraceptive services. Further work should clarify the role of vouchers in empowering WLWH to avoid unintended pregnancies over time.

**Trial registration:**

ClinicalTrials.gov NCT02964169.

## Abstract 2

Esther Cathyln Atukunda and colleagues reveal the uptake of contraceptive care for postpartum women living with HIV when offered family planning vouchers.

## Author summary

**Why was this study done?:**

- Unwanted pregnancies remain a burden for women living with HIV (WLWH).
- Family planning prevents unplanned pregnancies while promoting longer birth intervals, key strategies to eliminate perinatal transmission of HIV and promote maternal and child health.
- To date, previous studies have not compared whether family planning vouchers are as effective in driving differences in modern contraceptive method uptake or discontinuation by a common follow-up point postpartum. The studies also do not report on WLWH enrolled immediately postpartum in a public health facility and subject to standard limitations of public sector healthcare facilities in the region.

**What did the researchers do and find?:**

- We performed a randomized controlled trial between October, 2016 and June, 2018 at a referral hospital in southwestern Uganda. This interim analysis included adult WLWH randomized and enrolled equally to receive a family planning voucher or standard of care (control). We intended to document the rate and time of initiation, continuation, and change of a modern family planning method postpartum.
- By 8 weeks postpartum, more women initiated family planning in the voucher group (144 [91%]) than in the control group (83 [52%]). The family planning uptake rates were also high for both groups, with higher rates among the intervention group at 12, 14, 16, and 20 weeks postpartum.
- The average time to family planning initiation was substantially reduced from 9.3 weeks to 5.9 weeks for those in the voucher compared to control arms, respectively.
- Method mix did not differ by group: injectables were selected by most women (50%), for which 52% of this proportion were in the experimental arm. The proportion of women using implants was 20% (N = 60), with more women (60% versus 40%) opting for implants in the control arm than in the experimental arm. Less than 10% in each arm selected condoms, oral contraception, or IUDs.
- Similar proportions of women changed contraceptive methods over the 6-month follow-up period in the voucher and control groups. More women in the control group discontinued contraception for 1 to 2 weeks or more than 4 weeks compared to those given a family planning voucher.

**What do these findings mean?:**

- These findings indicate that a well-structured, time-bound family planning voucher program could increase early postpartum contraceptive uptake and continuation in a setting in which users are faced with financial, knowledge, and structural barriers to contraceptive services.
- The increased family planning uptake observed in this study may have been due to improved availability of contraceptives at the referral hospital, plus one-on-one postpartum counseling provided by a well-trained nurse as recommended versus routine care practiced at similar public facilities.
- Further work needs to clarify the role of vouchers in empowering WLWH to avoid unintended pregnancies over time.

## Introduction

HIV status and the availability of ART influence the desire and expectations to have children among women living with HIV (WLWH) [1, 2]. However, unwanted pregnancies remain a burden for WLWH. As many as 85% of pregnancies occurring within 3 years following ART initiation were reported as unwanted among African WLWH in Abidjan [3]. Family planning prevents unplanned pregnancies while promoting longer birth intervals, key strategies to eliminate mother-to-child transmission of HIV and promote maternal and child health [4–7]. Overall, modern contraceptive prevalence for married and unmarried women in Uganda is 35% and 51%, respectively [8]. The prevalence is 45% for WLWH [1]. Unmet contraceptive need exposes WLWH and their families to increased risks of unwanted/unplanned pregnancies, perinatal HIV transmission, and pregnancy complications.

In Uganda, over 90% and 40% of postpartum WLWH want to delay or avoid pregnancies for the next 1 and 2 years, respectively [9]. However, utilization of prepregnancy family planning among WLWH remains low, with over 50% of women on modern contraception using condoms as the only method of family planning [1]. Although conception can occur as early as 25 days after giving birth, especially if women are not regularly breastfeeding [7, 10], family planning may not be at the top of the couple’s priority list of concerns, with most couples resuming sexual activity before family planning methods are initiated [5, 11].

Family planning methods are widely available in most public facilities in Uganda, albeit with reported stockouts of some contraceptive methods, particularly the long-acting methods, leading to unwanted pregnancies [12]. Women at risk of or with a history of physical and sexual violence and poor birth outcomes (preterm birth, infant death, stillbirth, low birthweight, complicated pregnancy) have low utilization of postpartum contraception [11]. The data in these studies suggest uptake increases with increased preventive screening, contraceptive awareness, availability, and/or coverage, especially when integrated into routine healthcare. Structured counseling before discharge in an inpatient setting also increases modern contraceptive method uptake among postpartum women [13]. Other studies in lower-resourced settings demonstrated that providing vouchers to women individually and/or to their spouses to facilitate free access to family planning services improves contraceptive uptake and use [14, 15]. These studies suggest that a subsidized or free voucher may be used to support women to initiate family planning in lower-resourced settings amid health facility challenges. However, these studies do not compare whether vouchers are as effective in driving differences in modern contraceptive method uptake or discontinuation by a common follow-up point postpartum. The studies also do not report on WLWH enrolled immediately postpartum in a public health facility and subject to the standard limitations of public sector healthcare facilities in the region.

We performed a randomized controlled trial aimed at providing information on whether a family planning voucher, inclusive of structured immediate postpartum counseling, has a measurable impact on early initiation and consistent uptake of contraceptive methods among recently postpartum WLWH delivering at a publicly funded regional referral hospital in rural, southwestern Uganda. We hypothesized that improved family planning support through a focused voucher would increase early contraception uptake among WLWH. We conducted an interim analysis of the first phase of a family planning support intervention to delineate effects of each phase of this intervention within the parent trial, aimed at assessing effective use of contraception at 12 months postpartum.

## Methods

### Study design and setting

This analysis includes preliminary data collected from WLWH enrolling in a randomized controlled trial at the maternity ward of a regional referral hospital in southwestern Uganda. Mbarara Regional Referral Hospital (MRRH) is a publicly funded teaching hospital serving 10 districts with a population of over 5 million people. The parent trial aims to evaluate the effect of family planning support versus standard of care on contraceptive use at 12 months postpartum (NCT02964169). Here, we present interim data up to 6 months postpartum. The hospital is equipped with trained staff, midwives, and obstetricians able to offer comprehensive family planning services. Women accessing care at this hospital represent varied social and demographic backgrounds. The hospital performs over 12,000 deliveries annually and reports a 13% HIV prevalence among women (hospital records).

### Participants and recruitment

This study was initiated in October, 2016, and enrollment ended in May, 2017. Eligible participants were WLWH ≥ 18 years of age admitted to a postnatal ward within 5 days postpartum regardless of pregnancy outcome and qualified for any family planning methods available. The exclusion criteria included 1) HIV negative, 2) history of hypersensitivity to latex, 3) no male sexual partner and/or not anticipating one for the next 2 years, 4) only sexual partner has had vasectomy, 5) resides and works more than 20 km from the study site, and 6) inability to complete informed consent process as assessed by the study nurses. Trained research assistants (RAs) approached WLWH in the postnatal ward within 12 hours after delivery to capture all women delivering at this facility. RAs obtained voluntary written informed consent from all eligible participants in the local language in a private area of the hospital. All consenting participants gave written informed consent or, for those who could not write, a thumbprint was made on the consent form. A primary partner was defined either as a regular spouse who is also a regular sexual partner or the most recent sexual partner if no main partner was named.

### Study procedure

Because of structural and capacity challenges at the referent hospital site, routine discharge is often completed without family planning counseling.

**Fig 1:**
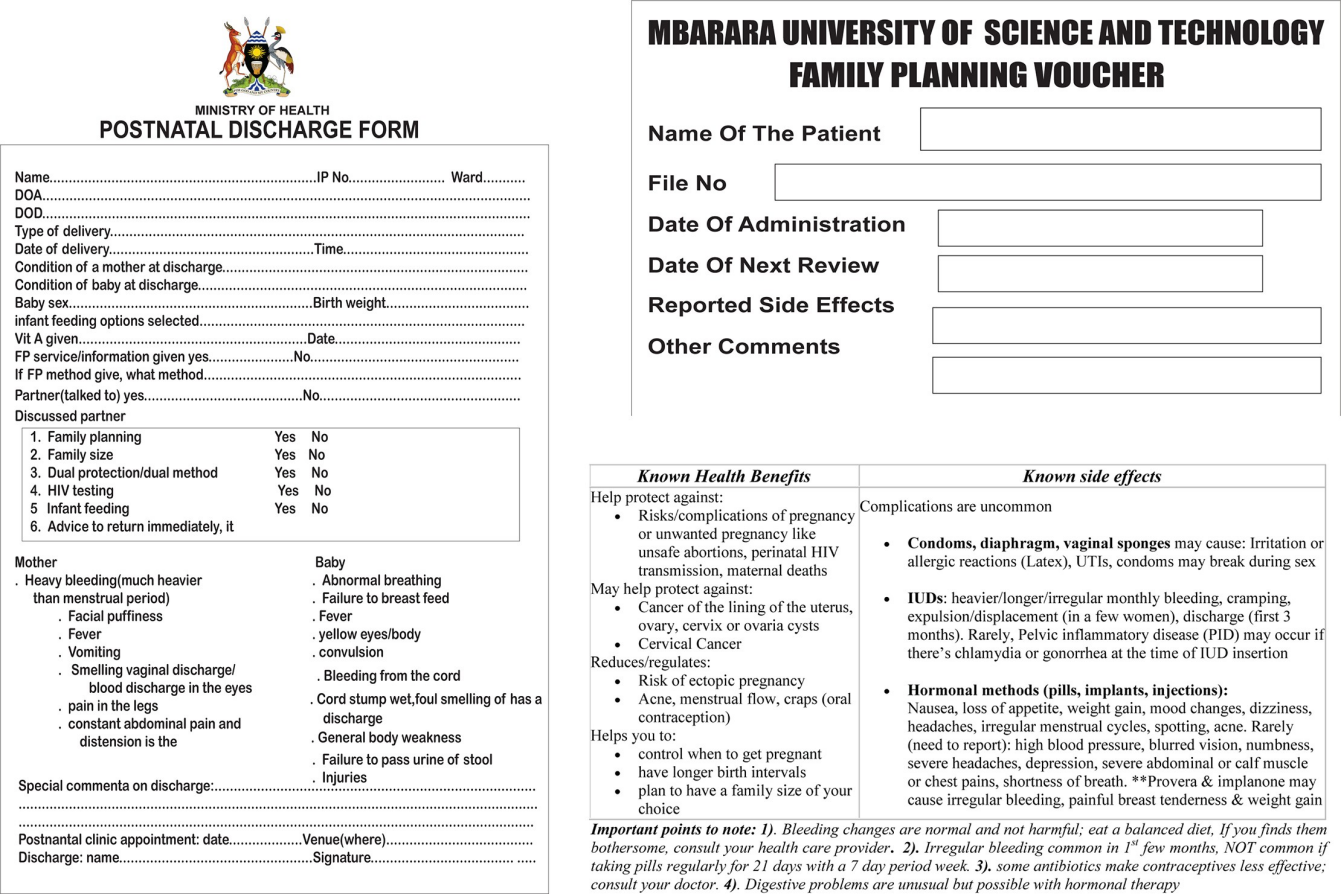
Ministry of health discharge form and the family planning voucher. DOA, Date of Admission; DOD, Date of Discharge; FP, family planning; IP, In-patient; IUD, intrauterine device; PID, Pelvic inflammatory disease; UTI, Urinary Tract Infection.

1. Family planning voucher intervention: following delivery, the women randomized to the intervention group were counseled and given a family planning voucher by the study nurse. Our one-on-one educational counseling was semistructured, providing face-to-face standardized (a list of items to talk about was generated) information to the woman and available partner on the available contraceptive methods, family size, medical eligibility for the different contraceptive methods, dual contraception, when to start contraception, how to use the contraceptives, potential side effects and benefits/effectiveness, and where the different family planning methods can be accessed (Fig 1). Structured, immediate postpartum counseling was offered in a clinic setting in a private room by a well-trained study nurse and lasted up to 40 minutes (S1 Text). All women were given opportunity to ask questions to facilitate women’s informed choice of any of the 5 freely available family planning methods at MRRH (both long- and short-acting modern contraceptive methods, including condoms, injectables, contraceptive pills, copper IUDs, and contraceptive implants). WLWH were also counseled on the standard days method (SDM) and lactational amenorrhea method (LAM). The same voucher and counseling was also given to the male sexual partner, when available, because of its identified effect on family planning utilization [15]. The voucher was used as an incentive to motivate women to seek/demand and access family planning easily at family planning clinics. A well-trained nurse was available at the postnatal clinic to identify women with vouchers to access the relevant service provider within 1 hour of arrival. Waiting time to see a service provider at the clinic was reduced to 1 hour maximum.

Although family planning is freely available in public health facilities, stockouts, especially of the long-acting contraceptive methods (implants and IUDs), attributed mainly to supply chain challenges are common [12]. The study promoted minimal stockouts of all methods at MRRH during the study period through regular involvement in forecasting and ordering. Private facilities rarely experience contraceptive method stockouts [12], and thus the family planning voucher also offered an opportunity for free administration (for example, injection, IUD placement, implant) of a contraceptive method purchased outside of the public healthcare facility. For this study, women who reside and/or work within 20 km from MRRH were enrolled, and thus all women were in close proximity to a facility with family planning services.

The voucher was offered for free and had an expiration of 3 months from the date of delivery. The voucher included detailed information about side effects for the different contraceptive methods as well as a general overview on benefits of family planning (Fig 1). Within 3 months postpartum, the women were expected to have returned to a health facility for at least two of their scheduled routine postnatal visits and/or immunization appointments.

2. Control group: in order to align the control group with guidelines-based standard of care, women in the control group were offered routine family planning counseling at discharge as defined by the Uganda clinical guidelines [16] by a well-trained ward nurse and documented in the Ministry of Health discharge form (Fig 1). The control group was not given a voucher.

Women from both groups were invited to start any available family planning method prior to discharge. The choice and place of family planning was entirely up to the participants regardless of group. All women accessing services at the hospital family planning clinic received care by a well-trained dedicated nurse to counsel and administer a chosen family planning method. Permission to contact spouses/sexual partners was obtained from all enrolled women. If permission to contact was given, a spouse/sexual partner was contacted, enrolled, and interviewed at baseline and 6 months for both groups. The spouses for controls were not given vouchers (S2 Text).

### Randomization and blinding

A study biostatistician generated a randomization list with a block size of 20, totaling 160 participants equally in each of the two groups into which mothers could be randomly assigned and enrolled. The aim of the study and details of the procedures to be involved in the trial, were explained before randomization. Once mothers consented to participate in the study, a study number was allocated by the research assistant by taking the next in a series of similar opaque envelopes provided to conceal allocation of groups. These opaque envelopes were labeled with a computer-generated list of numbers with group allocation. The RAs were blinded to the group allocation until eligibility and study participation was confirmed. They were also blinded to the hypothesis of the study.

WLWH were screened for eligibility and enrolled equally into the intervention arm (family planning support) and standard of care (control group) between October, 2016 and May, 2017. Women were followed for 1 year. All participants completed interviewer-administered interviews at baseline and 6 months postpartum. Interviews were conducted by two trained RAs fluent in English and the main local language in a private office space. Each interview took about 30–45 minutes. A different RA from the one enrolling participants was trained to specifically collect follow-up data to limit social desirability bias. Data were collected electronically. The data analyst was blinded to the group allocated to different study participants. A transport refund of US$3 was given on each visit.

### Study measures

A blood sample was drawn at baseline to confirm HIV status and measure CD4 cell count. A structured face-to-face questionnaire was completed at enrollment to collect information on sociodemographics; depression and health[17]; reproductive history; partnership dynamics (for example, HIV serostatus disclosure, partner HIV serostatus); perception, use, and knowledge of contraception; decision-making [18–23]; food insecurity [24, 25]; alcohol use in the last year [26]; HIV stigma [27]; social support [28]; and pregnancy intentions or aspiration [29–31].

### Study outcome

Our primary outcome of interest for this interim data was initiation of a modern family planning method within 8 weeks postpartum (the primary outcome for the full trial is effective contraceptive use at 12 months postpartum). Initiation of a modern family planning method at any facility of choice within 8 weeks postpartum was by both self-report and reviewing the participant’s postnatal chart by the study RA to identify and confirm initiation of family planning regardless of where the service was obtained. Outcomes from both reports were evaluated to confirm the internal validity and consistency of the two measures. Secondary outcomes included pregnancy incidence, mean time without contraception and family planning initiation at 12, 14, 16, and 20 weeks postpartum, and family planning discontinuation and/or change. Although postpartum counseling on contraceptive methods focused on the 5 methods—condoms, injectables, contraceptive pills (including progestin-only pills for breastfeeding mothers), copper IUDs, and contraceptive implants—as provided at MRRH, modern family planning was defined as use of these 5 and any other methods such as a diaphragm or cervical cap that participants could have obtained from other facilities.

### Sample size and statistical analysis

One-third of the pregnant WLWH in Uganda report an unmet need for contraception services and family planning support [32]. Provision of a family planning voucher has a significant impact on contraceptive uptake and long-term contraceptive use by an increase of 18 percentage points within 2 years of the reporting period among postpartum women [15]. The current contraceptive uptake among WLWH is 45% [1]. We therefore hypothesized that improved and focused family planning support through a voucher will increase contraceptive uptake among HIV-positive postpartum women to at least 63% within a follow-up period of 1 year postpartum. Allowing for a two-sided type I error of 5%, our target sample size was 320 postpartum women (with equal numbers of participants in the intervention and control groups) to enable 90% power to demonstrate a significant difference between groups.

We described demographic and clinical data for the cohort using standard descriptive statistics. The Household Food Insecurity Access Scale (HFIAS) was calculated as recommended [33]. We compared dichotomous outcomes between study groups by estimating crude odds ratios (ORs) with 95% confidence intervals, and testing for differences between the two groups. We estimated *P*-values with chi-squared testing using a level of significance of 0.05. We compared continuous outcomes and estimated *P*-values using Student *t* tests. All primary and secondary outcomes were analyzed using intention-to-treat analyses (although no participants were misallocated a group [23]). Although our study was fully randomized, the differences in baseline characteristics noted between study groups was assessed for confounding by fitting multivariable logistic regression models. As per the revised CONSORT guidelines for reporting randomized trials [26], we assessed for subgroup effects for the following characteristics by testing the significance of interaction terms in a multivariable regression model: 1) children living in household below 18 years of age (dichotomized into <3 children and ≥3 children categories), 2) parity (dichotomized into 1–3 and >3), 3) prenatal visits (<3 and ≥3 visits), 4) household income (≤150,000 and >150,000 Ugandan shillings), 5) involvement in any domestic violence (involvement, no involvement), 6) religion (Catholic, Protestant, others), and 7) duration on ART (<4, 4–8, and >8 years). A Mantel–Haenszel test was also done to control for each of these variables. All statistical analyses were performed using STATA version 13.0 (Statacorp, College Station, TX, USA).

### Compliance with ethical standards

All human subjects’ ethical approvals were obtained from Institutional Review Committees of Mbarara University of Science and Technology and Uganda National Council of Science and Technology and registered with clinicaltrials.gov (NCT02964169). A research assistant trained in human participant research conducted informed consent procedures with eligible participants in the local language in a private area. Written informed consent was obtained from all eligible participants.

## Results

**Fig 2:**
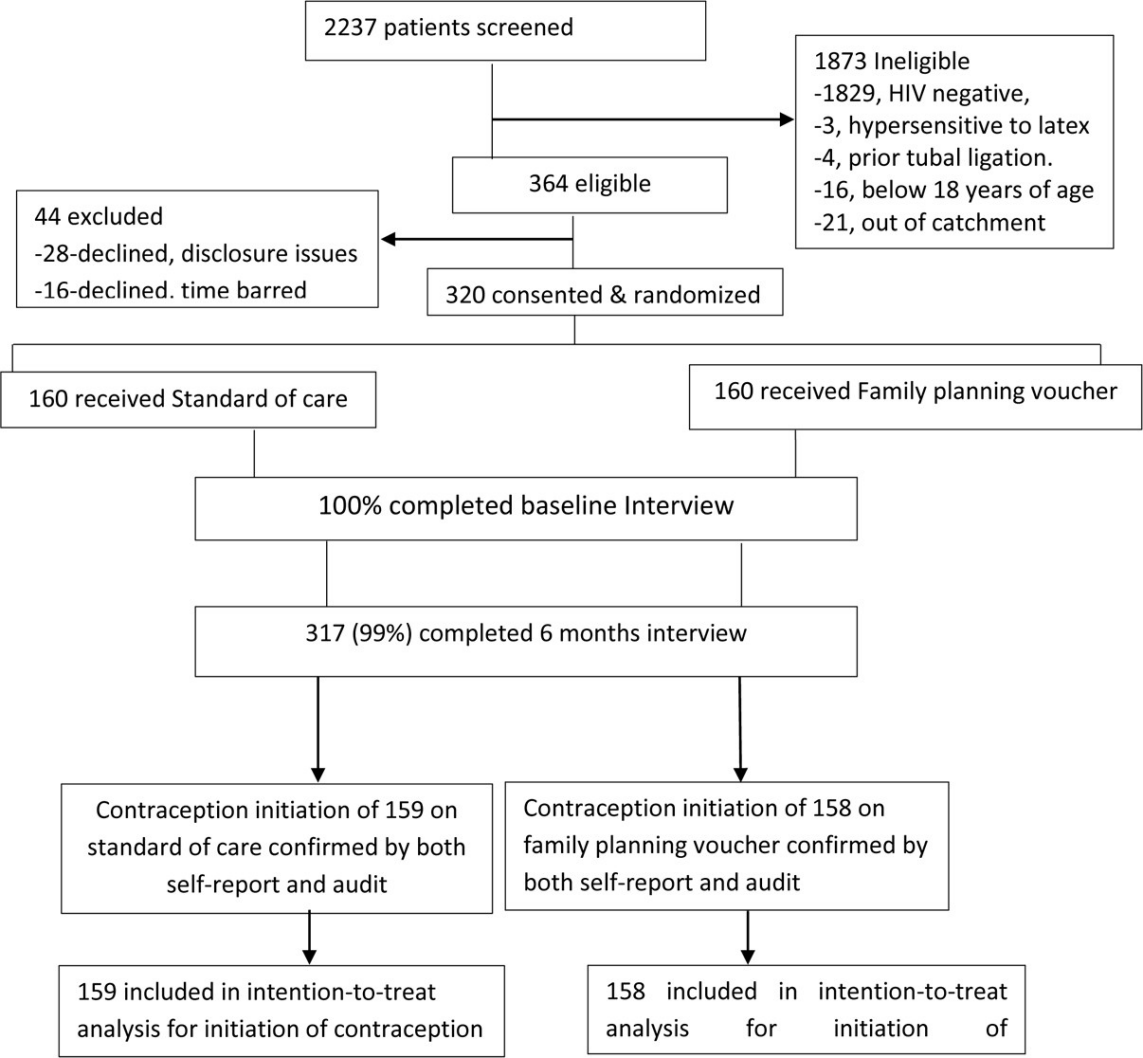
The trial profile.

Of the 2,237 women screened for eligibility between October, 2016 and May, 2017, 364 participants were eligible (Fig 2). A total of 28 women declined participation because of partner disclosure issues, and 16 declined because of the time commitment. 1,873 women were excluded because of HIV-negative serostatus (1,829), residing and working outside catchment area [21], age below 18 years [16], history of tubal ligation [4], or reported history of hypersensitivity to latex [3]. A total of 320 postpartum WLWH were randomized and enrolled equally into the family planning voucher and control arms of the study following delivery at MRRH. Three hundred and seventeen (99%) of enrolled participants completed all study procedures.

The mean age of participants was 29.6 (SD = 6.0) and 30.0 (SD = 5.9) years for the standard of care and family planning voucher groups, respectively. Mean CD4 count was 396 cells/mm^3^ (SD = 61) for those enrolled in the control group versus 393 cells/mm^3^ (SD = 64) in the family planning voucher group. At enrollment, half of the women in both the voucher (N = 87, 55%) and control (N = 86, 54%) groups wanted to have a child in 2 years postpartum. Over 80% of referent pregnancies in the voucher (N = 136, 86%) and control (N = 128, 81%) groups were planned. All women were accessing ART, with mean ART duration of 5.1 years (SD = 4.5) for those in the voucher group and 4.1 years (SD = 3.3) for those enrolled in the control group. Almost half of participants (46%) attained education greater than primary (50% versus 43%). A small number of male sexual partners participated in the study, including 18 (11%) and 21 (13%) for the voucher and control, respectively. Most of the women (N = 107, 70% versus N = 103, 69%) reported prior use of modern family planning methods. None of the women opted to start or receive immediate postpartum family planning before discharge. Other demographic and clinical characteristics were similar between the two groups, as presented in Table 1.

**Table 1:** Baseline demographic and clinical characteristics of recently postpartum WLWH in Uganda, N = 317.

Characteristics	Standard Care (n = 159)	FP Voucher (n = 158)
	Mean (SD) or n (%)	
Mean age (years)	29.6 (6.0)	30.0 (5.9)
Partner age (years)	34.4 (7.2)	34.9 (7.3)
Mean CD4 (SD)	396 (61)	393 (64)
Partner living with HIV	111 (69.8)	108 (68.4)
Mean duration on ART (years)	4.1 (3.3)	5.1 (4.5)
Duration on ART (years):		
<4	69 (54.3)	63 (50.4)
4–8	40 (31.5)	39 (31.2)
>8	18 (13.2)	23 (18.4)
Educational attainment greater than primary	68 (42.8)	79 (50.0)
Sexual partner contacted and enrolled in study	21 (13.2)	18 (11.4)
Religion:		
Catholic	41 (26.1)	25 (16.1)
Protestant	90 (56.6)	100 (63.3)
Others	26 (16.6)	30 (19.4)
Household children <18 years:		
0–1	45 (28.3)	45 (28.5)
2–3	92 (58.9)	75 (47.5)
≥4	22 (13.8)	38 (24.1)
Ever used any modern contraception in last 10 years	107 (69.5)	103 (68.7)
Used modern contraception in 2 years prepregnancy	61 (37.7)	60 (38.0)
Desire for pregnancy in 2 years	87 (54.7)	86 (54.4)
Desire for pregnancy in 1 year	5 (3.5)	4 (2.6)
Partner desires another child in 2 years	94 (59.1)	90 (57.0)
Most recent pregnancy planned	136 (85.5)	128 (81.0)
Parity:		
1	25 (15.7)	28 (17.2)
2–3	92 (57.9)	71 (44.9)
>3	42 (26.4)	59 (37.3)
Prenatal visits attended:		
0–1	6 (3.8)	4 (2.5)
2–4	117 (73.6)	125 (79.1)
>4	36 (22.6)	29 (18.4)
Severe food insecurity^a^	30 (18.9)	31 (19.6)
Depression score^b^	4.2 (2.8)	4.6 (3.8)
Consumed alcohol in last year	108(67.9)	106 (67.1)
Household income^c^:		
<100,000	91 (57.2)	74 (46.8)
100,000–150,000	31 (19.5)	31 (19.6)
>150,000	37 (23.3)	53 (33.5)
Monogamous household	128 (80.5)	135 (85.4)
Vaginal mode of delivery for last pregnancy	125 (78.6)	126 (79.8)
Domestic violence in any relationship	18 (14.4)	30 (23.8)
Disclosed HIV serostatus to sexual partner	133 (83.6)	138 (87.3)
Knows/sure about sexual partner’s serostatus	112 (70.4)	110 (69.6)
Takes part in decision-making	87 (54.7)	90 (57.0)
Opted to receive immediate postpartum FP before discharge	0 (0)	0 (0)

^a^HFIAS > 8 means severe food insecurity.

^b^This score ranges from 1–48, indicating 0 as no depression.

^c^As measured in Ugandan shillings; 1 USD = 3,650 Ugandan shillings.

**Abbreviations:** FP, family planning; HFIAS, Household Food Insecurity Access Scale; US, US dollar; WLWH, women living with HIV.

Both self-report and postnatal chart/record audit generated identical outcomes, confirming the internal validity and consistency of the two measures. By 8 weeks postpartum (primary outcome), family planning was initiated in 144 (91%) participants in the family planning voucher group and 83 (52%) participants in the standard of care group (OR 9.42; CI 4.67–13.97; Table 2). Contraceptive use rates continued to increase between 8 and 20 weeks and remained consistently significantly higher among the intervention group. The average time without contraception was reduced to 5.9 weeks postpartum (SD = 2.4) for those in the voucher arm compared to 9.3 weeks (SD = 5) in the control (*P* < 0.001). Approximately 10% of women (9.4% versus 2.5%) did not access contraception by 6 months postpartum.

**Table 2:** Primary and secondary outcomes by treatment group.

Outcomes	Routine Care (n = 159)	FP Voucher (n = 158)	OR (95% CI)	*P*-Value
**Primary outcome**				
Initiation of FP:				
8 weeks postpartum	83 (52.2%)	144 (91.4%)	9.42 [4.67–18.97]	<0.001
**Secondary outcomes**				
Initiation of FP:				
12 weeks	115 (72.3%)	148 (93.7%)	5.66 [2.65–12.12]	<0.001
14 weeks	122 (76.7%)	149 (94.3%)	5.02 [2.27–11.10]	<0.001
16 weeks	134 (84.3%)	151 (95.6%)	4.02 [1.66–9.77]	0.001
20 weeks	139 (87.4%)	152 (96.2%)	3.65 [1.40–9.47]	0.004
Initiation of contraception for women that wanted no pregnancy in the next 2 years:^a^				
8 weeks postpartum	38 (52.8%)^b^	65 (90.3%)^b^	8.31 [3.06–22.59]	<0.001
6 months postpartum	65 (90.3%)	70 (97.2%)	4.01 [1.28–12.54]	0.010
Mean (SD)	9.3 (5.0)	5.9 (2.4)	N/A	<0.001
Enrolled on contraception by 6 months	144 (90.6%)	154 (97.5%)	4.01 [1.28–12.54]	0.010
Pregnancy by 6 months postpartum	1 (0.6%)	0 (0%)	0.00	0.301
Changed contraception in 6 months^c^	5 (3.5%)	8 (5.2%)	1.52 [0.49–4.78]	0.468
Discontinued family planning^c^:				
1–2 weeks	19 (13.2%)	07 (4.6%)	0.31 [0.13–0.78]	0.008
≥4 weeks	15 (10.4%)	03 (2.0%)	0.17 [0.05–0.62]	0.002
Reasons for change/discontinuation:				
Wanting a child/death of a child	2 (1.4%)	2 (1.3%)	0.93 [0.13–6.74]	0.946
No longer needing protection	5 (3.5%)	3 (2.0%)	0.55 [0.13–2.36]	0.417
Method-related side effects	17 (11.8%)	5 (3.3%)	0.25 [0.09–0.71]	0.005

^a^Excludes those intending to have pregnancy in the next 2 years.

^b^N = 72.

^c^Excludes those not enrolled in FP.

**Abbreviations:** FP, family planning; N/A, not applicable; OR, odds ratio.

**Fig 3:**
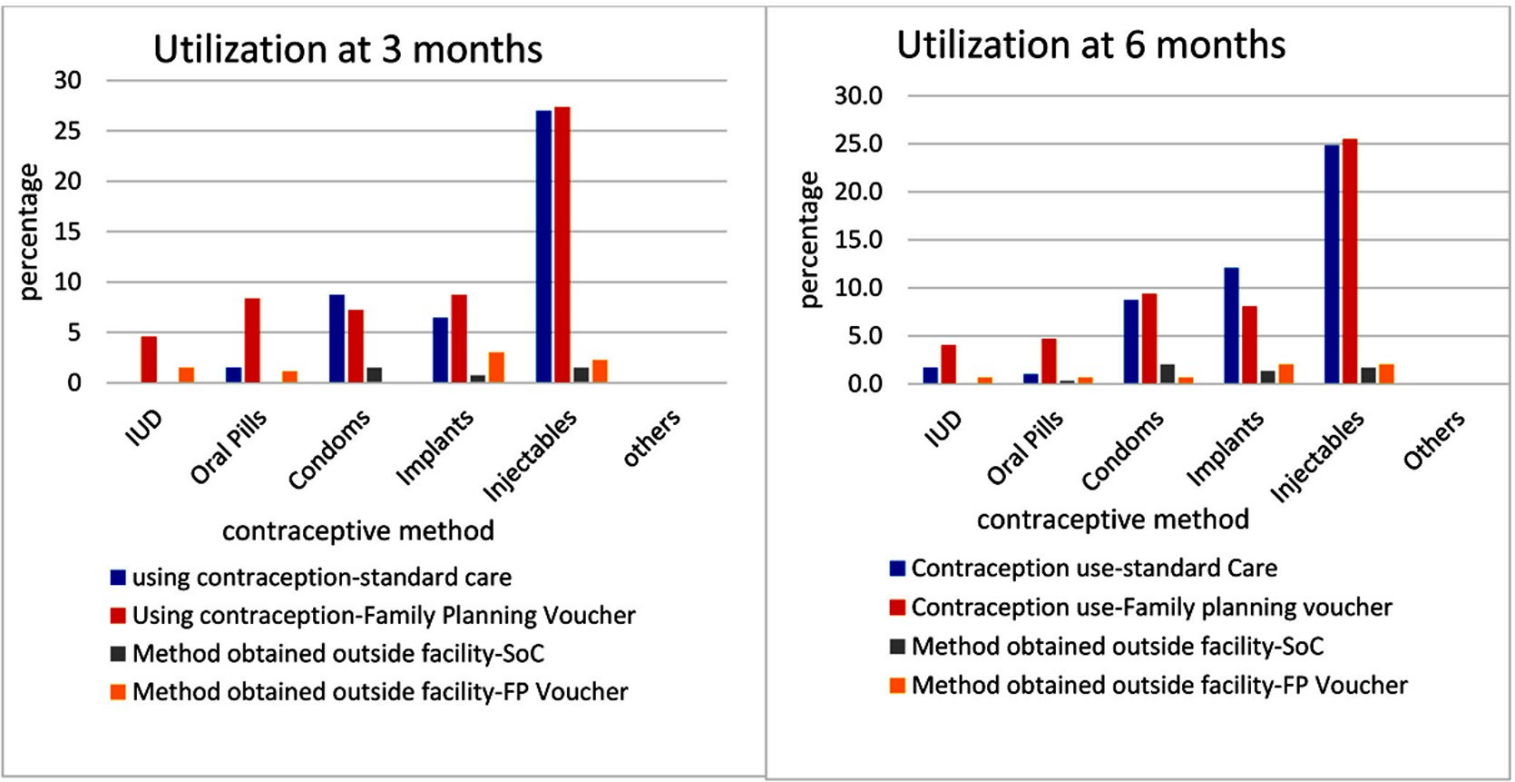
Percentage utilization of family planning methods at 3 months (N = 263) versus 6 months (N = 298) postpartum by study group. FP, family planning; SoC, Standard of care.

One pregnancy was reported in the group receiving standard of care; none were reported in the voucher group. Of those enrolled on family planning by 6 months (N = 154, 98% versus N = 144, 91%, *P* = 0.010 in the voucher and control groups, respectively), method mix did not differ by group: injectables were selected by most women (N = 150, 50%), and 52% of this proportion were in the experimental arm versus 48% in the control arm. The proportion of women using implants followed at 20% (N = 60, 40% versus 60%), with <10% in each arm selecting condoms, oral contraception, and IUDs (Fig 3). Most women enrolled on long-acting contraception initiated the method by 3 months postpartum (Fig 3). Availability of progesterone-only pills increased their demand and uptake by 3 months postpartum, but most users opted to change to other methods by 6 months postpartum. No women reported use of a diaphragm or cervical cap. A few women (11%; 6% versus 5%) obtained a contraception method from a health facility other than MRRH. Although no differences were detected, more women in the voucher group changed contraceptive method within the course of 6 months (N = 8, 5% versus N = 5, 4%, *P* = 0.467), all of those women with switches opted for long-acting implants or IUD from a less effective contraceptive method (condoms). Importantly more women in the control group discontinued contraception for 1 to 2 weeks (N = 19, 13% versus N = 07, 5%, *P* = 0.008) or more than 4 weeks (N = 15, 10% versus N = 3, 2%, *P* = 0.002) compared to those given a family planning voucher within the 6-month postpartum period. Of the 144 (45%) who did not want any pregnancy in the next 2 years, 65 (90%) and 70 (97%) initiated family planning in the voucher group versus 32 (53%) and 65 (90%) in the control group by 8 weeks and 6 months, respectively.

While we performed a randomized control trial and anticipated that any differences in baseline characteristics occurred by chance, we detected baseline differences in children living in a specific household, parity, prenatal visits, household income, domestic violence, religion, and duration on ART (Table 1). We assessed whether the estimated OR was affected by differences in baseline characteristics between groups by fitting multivariable logistic regression models. In these models, we found no meaningful change in the OR of family planning initiation at 8 weeks postpartum for intervention versus control participants after adjustment for the factors listed above (adjusted OR 10.47; 95% CI 4.99–21.99; *P* < 0.001). In stratified analyses to assess for differences in our primary outcome within subgroups (parity, children <18 years of age in a household, religion, age, domestic violence, ART duration, household income, previous use of contraception), no subgroup-by-treatment interaction terms were significant (Table 3). Thus, while we never set out to estimate effects within subgroups, our results do not suggest differential effects in treatment within specific subgroups of WLWH.

**Table 3:** Maternal baseline subcategories by study arm with initiation to FP at 8 weeks postpartum.

Subgroup (n)	Routine Care (n = 159)	FP Voucher (n = 158)	OR (95% CI)	*P*-Value	Adjusted OR	*P*-Value	*P*-Value for Interaction Term
**Children <18 years**							
<3	48/101 (47.5%)	73/81 (90.1%)	10.08 [3.98–25.5]	<0.001	1.00		
≥3	35/58 (60.3%)	71/77 (92.2%)	7.78 [2.66–22.70]	<0.001	9.10 [4.51–18.36]	<0.001	0.169
**Age (years)**							
18–29	44/80 (55.0%)	64/72 (88.9%)	6.55 [2.59–16.53]	<0.001	1.00		
≥30	39/79 (49.4%)	80/86 (93.0%)	13.68 [4.65–40.21]	<0.001	9.25 [4.62–18.52]	<0.001	0.576
**Parity**							
<3	61/117 (52.1%)	88/99 (88.9%)	7.34 [3.33–16.19]	<0.001	1.00		
≥3	22/42 (52.4%)	56/59 (94.9%)	16.97 [3.71–17.59]	<0.001	9.02 [4.51–18.01]	<0.001	0.978
**Prenatal visits**							
<4	38/64 (59.4%)	39/40 (97.5%)	6.00 [1.67–21.52]	<0.001	1.00		
≥4	45/95 (47.4%)	105/118 (89.0%)	11.13 [4.75–26.16]	<0.001	9.40 [4.64–19.02]	<0.001	0.138
**Household income**							
≤150,000	63/122 (51.6%)	95/10 (90.5%)	8.90 [3.92–20.21]	<0.001	1.00		
>150,000	20/37 (54.1%)	49/53 (92.5%)	10.41 [2.68–40.4]	<0.001	9.26 [4.59–18.68]	<0.001	0.797
**Domestic violence**							
No	57/107 (53.3%)	88/96 (91.7%)	9.65 [3.90–23.85]	<0.001	1.00		
Yes	6/18 (33.3%)	25/30 (83.3%)	10.00 [2.00–49.91]	<0.001	9.73 [4.42–21.42]	<0.001	0.124
**Religion**							
Catholic	18/41 (43.9%)	20/25 (80.0%)	5.11 [1.47–17.80]	0.004	1.00		
Protestant	53/90 (58.9%)	95/100 (95.0%)	10.50 [2.06–53.55]	<0.001	9.28 [4.52–19.03]	<0.001	0.183
Others	12/26 (46.2%)	27/30 (90.0%)	13.26 [4.39–40.04]	<0.001			
**ART duration (years)**							
<4	36/69 (52.2%)	58/63 (92.1%)	10.45 [3.31–32.96]	<0.001	1.00		
4–8	21/40 (52.5%)	34/39 (87.2%)	6.15 [1.81–20.90]	<0.001	8.28 [3.92–17.49]	<0.001	0.540
>8	8/18 (44.4%)	20/23 (87.0%)	8.33 [1.47–47.31]	0.004			
**Ever used modern contraception**							
No	26/47 (55.3%)	39/47 (83.0%)	3.94 [1.44–10.75]	0.004	1.00		
Yes	57/107 (53.3%)	98/103 (95.2%)	17.19 [5.65–52.28]	<0.001	8.98 [4.39–18.37]	<0.001	0.814

**Abbreviations:** FP, family planning; OR, odds ratio.

## Discussion

In this study, we found that improved support to postpartum WLWH through a time-bound health-worker–supported family planning voucher increased early contraceptive uptake at a public regional referral hospital in southwestern Uganda. We found that over 90% of women supported through this family planning voucher accessed family planning by 8 weeks postpartum, which corresponds to 9.42 (CI 4.67–13.97) increased odds of initiating family planning by 8 weeks postpartum. This voucher approach substantially reduced the number of weeks for women to access family planning: 5.9 (SD = 2.4) versus 9.3 (SD = 5.0) weeks. The rates of secondary outcomes, including family planning initiation by 12, 14, 16, and 20 weeks postpartum, remained significantly higher in the voucher group compared to standard of care. Family planning uptake at these later time points was high in both groups. While not powered to do so, we observed a significant difference in family planning discontinuation rates between groups, with women in the intervention less likely to discontinue contraception. Given the importance of contraception to support WLWH in having healthy pregnancies, eliminate Mother-to-child transmission of HIV (MTCT), and reduce maternal and child mortality, this study suggested the importance of integrating such a focused, health-worker–supported, and time-bound family planning voucher program into comprehensive reproductive healthcare for WLWH.

Prior studies demonstrated that subsidized or free reproductive health vouchers improve uptake of modern contraception among women in lower-resourced settings [14, 15, 34, 35]. A combination of a social franchise and family planning voucher program to provide family planning counseling and a broader contraceptive choice, inclusive of long-acting and permanent methods, increased contraception access and uptake in Ugandan hard-to-reach populations [36]. However, prior evidence has been limited by variability in designs and measured outcomes. Prior studies did not compare whether vouchers were equally as effective in driving differences in modern contraceptive method uptake by a common follow-up point, like 8, 12, 14, or 20 weeks postpartum, among women or WLWH in Uganda. Ashraf and colleagues (N = 1,516], for example, documented an increase in contraception use, particularly with long-acting contraceptive methods, among all women of child-bearing age given vouchers compared to those without. These prior studies also reported no minimum durations required for these voucher interventions to effect notable change in contraceptive uptake and/or initiation. This study was powered to detect a significant benefit of a comprehensive family planning voucher compared to standard of care in increasing early initiation of effective contraceptive methods among WLWH following childbirth.

The first postnatal visit is typically scheduled at 6 weeks postpartum. Our findings suggest a crucial role of a comprehensive time-bound, health-worker–supported family planning voucher that broadens client access to postnatal services and facilitates improved information transfer to demand and utilize different available contraceptive methods whenever needed or wanted. The voucher gave women flexibility to utilize postnatal clinic services at MRRH as and when needed within the same facility where the majority of women receive routine ART services. This flexibility facilitated additional early visits to routine postpartum care that may be desirable for earlier initiation of family planning [37]. Other scholars, however, have noted that up to a third of women starting a modern contraceptive method discontinue the method within the first year [38]. Another trial (N = 1,163) specifically comparing continuation rates and reasons for discontinuation of an intrauterine device in Pakistan showed no difference in discontinuation between the voucher and nonvoucher groups at 24 months [39, 40]. However, just like our study, the probability of continuation of an initiated contraceptive method was higher for the voucher cohort versus the nonvoucher cohort at 6 months. Although the inclusion/provision of information about family planning benefits and possible side effects on the voucher could have been helpful in continuously reminding women about expectations from the different contraceptive methods chosen [41], qualitative data analysis of interviews conducted with a subset of participants to understand how the voucher (information-on-voucher, counseling, time-bound, waiting time, methods administration) facilitated decision-making, initiation, and/or continuous use of contraception among these child-bearing WLWH is underway. Twelve-month postpartum data collection is still ongoing.

Our study supported all women in both arms throughout the study period to minimize stockouts of contraceptives through timely ordering and coordination of the routine supply chain. One-on-one family planning counseling was also provided to all postpartum women by a well-trained nurse as required, albeit structured for voucher holders, despite staffing challenges. Family planning uptake was therefore notably high at later time points for both groups. Interactive counseling helps women access important information to make informed decisions on the different available methods and improves acceptability, compliance, successful uptake, and continuation of contraceptive methods [42–44]. For example, social franchising used alongside free vouchers and counseling for long-term contraceptive choices significantly increased awareness of modern contraception by 5%, any use of modern contraception by 28.5%, and the overall contraceptive prevalence rate by 19.6% in rural areas of Pakistan [45]. In our study, we detected a significant difference of 39% of family planning initiation at 8 weeks postpartum between study groups. However, another study using a difference-in-difference design demonstrated that improved availability of modern contraceptives increased use of injectables and modern contraceptives by 350% and 50%, respectively [46]. Although we did not directly compare the use of long-acting versus short-acting contraceptive methods, our data support preferential uptake of implants (20%) and injectable contraception (50%) regardless of the study group among WLWH. A potential explanation for differences between our study and this prior data, which showed different effect sizes, is our exclusion of HIV-negative women and limiting the study to adults (18 years of age and above) residing and/or working within a catchment area of 20 km from MRRH. All women in this study were taking ART, with over 85% of them accessing their routine HIV care at MRRH, and reported a high rate of planned referent pregnancy (>80%). Our selection criteria therefore could have underestimated true differences in initiation of family planning specifically in the general population as opposed to WLWH engaged in regular healthcare. This exclusion may also have underplayed the effect of transport costs on contraception uptake, making this voucher even higher impact when locally accessible health facilities are technically and structurally ready to provide the required family planning services in terms of capacity and availability.

A few sexual partners participated in this study, which may have influenced reproductive decision-making for both study groups. The preference for concealable forms of contraception such as injectables and implants may have been attributed to the methods being seen as “easy to hide” and/or “less worrisome” compared to other contraceptive methods, especially for individuals experiencing intimate partner violence or relationships in which women were unable to make independent decisions regarding family planning [15, 47]. Concealable family planning methods may empower women to make their own healthcare decisions to access reproductive health services whenever financial and knowledge barriers are minimized [48]. Whereas many studies have documented the crucial importance of involving men in supporting women to access reproductive health services [9], the low engagement of men in this study did not deter women from accessing available family planning among more than 90% WLWH in either group by 6 months postpartum. Like other studies, the rate of women likely to use concealable injectable contraception diminished when women were enrolled to access family planning together with their spouses regardless of the study group.

The benefit of a voucher in facilitating early initiation of family planning in this trial was seen across all subgroups. Effect sizes, however, appeared bigger in certain subgroups, for example, women subscribing to a non-Catholic religion, those with parity more than 3, women aged 30 years and above, women who attended at least 4 prenatal visits, women with improved household income, and women with a previous history of modern family planning use, which corroborates prior work demonstrating higher rates in use of contraception among such populations [49–54]. This may also be corroborated with observed reduction in reported fertility aspirations among the same postpartum WLWH with increasing age, parity, and previous use of modern family planning methods [9]. Although there were observed differences in point estimates of family planning initiation in these subgroups, we found no significant differences in the effect of the intervention across these categories.

Our study had a number of strengths. This work presents preliminary follow-up data for a randomized controlled trial that started in October, 2016 and ended in June, 2018. The data analyst and the research assistant collecting follow-up data were blinded to study arm allocation. We performed this randomized controlled trial in a prototypical publicly funded and operated hospital in a rural setting with active postnatal and family planning clinics, subject to the standard limitations of public sector healthcare facilities in the region. The hospital carries out deliveries for over 12,000 women annually from various social and demographic backgrounds, making results generalizable to similar settings. Although a well-trained focused family planning nurse was identified from the same clinic and we directly facilitated her voluntarism with a small incentive of $30 per month within the study period, this has great potential for generalizability to similar publicly funded settings. The population of postpartum WLWH with a high proportion of contraception uptake also enabled us to document the differences in point estimates of when the family planning was initiated, change and discontinuation of the initiated contraceptive method, and the effect of baseline subcategories by study arm with initiation to family planning within a multivariate model. Another strength of our study was the observed low rate of eligible participants declining participation (N = 44, 12%). A review of stated reasons for declining participation revealed that most (N = 28, 64%) of participants who declined were worried about the study process leading to unintended disclosure of their status to their spouses, and the rest (N = 16, 36%) were uninterested in participating in a research study because of the time involved, which was perhaps not unexpected, given most women in the study (86%) had reportedly disclosed to at least one of their sexual partners. The exclusion rates were also low, and the study did not require partner participation, suggesting generalizability and replicability of our study findings.

Our study also had some limitations. We observed an increase in family planning uptake from about 38% from the prestudy period (as seen from baseline data) to over 90% in the study period for both groups, suggesting improved outcomes with good, one-on-one counseling as recommended by the standard of care guidelines versus routine care that is frequently practiced in similar publicly funded facilities. The use of a time-bound voucher with an expiration date of 3 months postpartum also could have encouraged use of the given vouchers within the limited time afforded. There may also have been the possibility of a Hawthorne effect, which might have resulted from improved availability of contraceptives at MRRH and use of a well-trained nurse to provide counseling and administration of these methods throughout the study period. The low rate of pregnancy may have been due to a shorter follow-up period afforded by these follow-up data. The general screening and enrollment of WLWH from the postnatal ward at MRRH might have introduced a selection bias towards relatively high-risk WLWH. However, this selection bias was minimized by ensuring availability of these contraceptive methods at the MRRH family planning clinic within easy reach of all women regardless of the group or involvement in the study.

### Conclusion and recommendations

We found that improved support of WLWH through a focused time-bound family planning voucher increases early postpartum contraceptive uptake at a publicly funded regional referral hospital in southwestern Uganda. Discontinuation from the initiated family planning method was low in our study population, albeit significantly less for the voucher group. There were similar rates of changes in the contraceptive method initiated and rates of reported pregnancy within the study period. These data demonstrate that, in settings where users are faced with financial, knowledge, and structural barriers to contraceptive services, a well-structured comprehensive family planning voucher program could facilitate early contraception uptake and continuation through improved counseling, continuous information transfer and education, and appropriate referral to centers where these services are freely offered. The dedicated one-on-one counseling and reviews on family planning seem to facilitate contextual understanding of key individual-level social–cultural, economic, demographic, and structural factors within which vulnerable couples or individuals understand, appreciate, and maximize the need to limit or delay births within a supportive environment, which can be adopted into routine HIV care accessible at all primary healthcare levels. This time-bound voucher intervention also seemed to expand client access to postnatal services and empower potential users, who seemingly and progressively generated an early demand through counseling to take up short- and long-term contraceptive methods as and when they needed or wanted them, especially “concealable” contraceptive methods, amid a noticeably low male involvement.

Further work should help to clarify the role of vouchers in empowering clients to avoid unintended pregnancies in the long term. Other ways of increasing male involvement in such family planning voucher programs should also be studied. Additionally, further evaluation of the actual and perceived barriers to modern family planning methods in resource-limited settings will help improve uptake and continuation of long-term effective family planning methods amid structural challenges and inconsistent availability and use in such settings.

## Supporting information

S1 Text Family planning voucher counseling standard operating procedure. (PDF)

S2 Text Study protocol. (PDF)

S3 Text Follow-up tool. (PDF)

S4 Text CONSORT checklist. (DOC)

## Abbreviations

DOA: Date of Admission
DOD: Date of Discharge
HFIAS: Household Food Insecurity Access Scale
IP: In-patient
IUD: Intrauterine Device
MRRH: Mbarara Regional Referral Hospital
MTCT: Mother-to-child transmission of HIV
LAM: lactational amenorrhea method
OR: odds ratio
PID: Pelvic inflammatory disease
RA: research assistant
SDM: standard days method
SoC: Standard of care
UTI: Urinary Tract Infection
WLWH: women living with HIV
