# Supplementary material for: Provision of family planning vouchers and early initiation of postpartum contraceptive use among women living with HIV in southwestern Uganda: A randomized controlled trial
Source: PLoS Med. 2019 Jun 21;16(6):e1002832. doi: 10.1371/journal.pmed.1002832 (PMC6588214; doi:10.1371/journal.pmed.1002832)
Supplement: S1 Text — (PDF) [file pmed.1002832.s001.pdf]

STUDY TITLE:	**The impact of a family planning support intervention on pregnancy desires and contraceptive use among recently postpartum HIV positive women delivering at MRRH**
**DOCUMENT**	**STANDARD OPERATING PROCEDURES FOR NURSE COUNSELLORS OFFERING FAMILY PLANNING COUNSELLING FOR VOUCHER HOLDERS**
**SOP Version No:**	**01**
**Date:**	**12TH July 2016**

Purpose of this counselling.

The primary objective of this family planning counselling is to help women living with HIV (WLWH) understand the role of family planning postpartum and help them decide voluntarily on the contraception method they wish to have. You can help them to choose a contraceptive method that is personally and medically appropriate. Through your counselling sessions with them, you will make sure that they understand the benefits and side effects of each method and how each of them is used correctly, to ensure informed decisions, safe and effective contraceptive use. Counselling will be done face-to-face in a private space provided next to the postnatal ward at MRRH.

Steps for conducting family planning counselling.

1. **Carefully prepare yourself to have good scientific knowledge of all the contraceptive** methods, and understand the practical part of family planning methods. You should be prepared to give an overview of the family planning methods and answer ALL questions comfortably and without embarrassment in relation to contraceptive myths, rumors, sexuality, infertility, sexually transmitted infections (STIs), HIV/AIDS, reproductive and personal concerns
2. ***Important to NOTE***:

- Please remember that family planning counselling is not a type of lecture from you to those who need your help
- Show utmost respect to the participant that you are counselling, and deal/respond to their problems/concerns about contraception in an honest straightforward way.
- Give and maintain the participant assurance of confidentiality while counselling
- ensure a private and quiet place provided next to the ward at all time
- Take sufficient time to explain and listen to the participant’s questions and concerns. Be empathetic at all time.
- Look out for myths and mis-information participants have about family planning and respectfully take time to provide accurate information.
- Find out participants’ views about contraception and child birth by encouraging them to talk. Avoid direct and judgmental questions such as: ‘Are you one of those people who believe that modern family planning is forbidden for religious people?’ Such questions sound critical and can make people feel inferior, or may make them mistrust you because they may ask themselves, ‘Why should I believe this person when all my relatives share my belief?’
- Use simple terminologies understood by the participants.

- Use the provided visual aids to explain the different contraceptive methods and avoid giving too much information to avoid confusion.
- Repeat the most important instructions about family planning again and again.
- Always test participants' understanding before leaving the room.
- Try to understand, and be sensitive to cultural and psychological factors that may affect participants in their communities from adopting and using family planning methods or on limiting child birth. ***Please note:*** Some methods may be unpopular with participants, for example a woman might not like the idea of having to insert a contraceptive into her body before having sex, or a man may think that a condom will take away the pleasure of sex.
- Some methods like cervical cap, may not be widely available in Uganda and others may not be regular and reliable. Please honestly discuss this with the participant, indicating other available alternative methods and where they can be accessed easily

3. **Carefully and with a lot of sensitivity to individual needs, use a step-by-step approach (GATHER) to remember to employ the 6 steps to help you offer effective family planning counselling.**

G Greet the participant respectfully.
A Ask them about their family planning needs.
T Tell them about different contraceptive options and methods.
H Help them to make decisions about choices of methods.
E Explain and demonstrate how to use the methods.
R Return/refer; schedule and carry out a return visit and follow up.

Note: ***Carefully define family planning in a manner that is well understood by the participant.***

4. **Provide method-specific counselling** by giving more information about the chosen method. Please explain the eligibility and instruct on how and when to use the method using the BRAIDED approach, and emphasize on the reminder information included on the voucher card. Please tell the participants when to return for follow-up, and ask them to repeat what you have said on key information.

BRAIDED APPROACH:

B Benefits of the method

R Risks of the method, including consequences of method failure
A Alternatives to the method (including abstinence and no method)
I Inquiries about the method (individual's right and responsibility to ask)
D Decision to withdraw from using the method, without penalty
E Explanation of the method chosen
D Documentation of the session for your own records.

5. **Schedule a convenient follow-up review date** that tallies with her postnatal visit, immunization visit or routine ART clinic to discuss/review concerns, questions and manage any problems and side effects related to the given contraceptive method, and to encourage to encourage the continued use of the chosen method if no problems are reported.

6. **Our check list**

**Key areas to engage before discharge**	**Check X if discussed**
Carefully define family planning	
Discuss family size	
Respectfully explain benefits/the need to start family planning/ delay child birth	
Discuss effectiveness of contraceptive methods	
Discuss available contraceptive methods, where to access them	
Medical eligibility for the different contraceptive methods	
Dual contraception	
When to start immediate postpartum contraception	
Demonstrate/discuss how to use methods	
Discuss potential side effects	
Check understanding	
Allow questions	
Repeat/summarise key information	
Explain information on the voucher	
Offer family planning of choice, or refer	
Provide review date	

7. DETAILS ON THE GATHER APPROACH

a) G-Greet the participant

- In the first case, give your full attention to your participants.
- Greet them in a respectful manner and introduce yourself after offering seats.
- Ask them how you can help them.
- Tell them that you will not tell others what they say.
- If the counselling is in a health institution, you have to explain what will happen during the visit, describing physical examinations and laboratory tests if necessary.
- Conduct counselling in a place where no-one can overhear your conversation.

b) A-Ask the participants about themselves

- Help them to talk about their needs, doubts, concerns and any questions they might have.
- If they are new, use a standard checklist or form from your Health Management Information system to write down their name and age, marital status, number of pregnancies, number of births, number of living children, current and past family planning use, and basic medical history.
- Explain that you are asking for this information in order to help you to provide appropriate information, so that they can choose the family planning method which is the best for them.
- Keep questions simple and brief, and look at her/them as you speak.
- Many people do not know the names of diseases or medical conditions. Ask them specific questions. Say, ‘Have you had any headaches in the past two weeks?’, or ‘Have you had any genital itching?’, or ‘Do you experience any pain when urinating?’ Do not say, ‘Have you had any diseases in the recent past’.
- If you have seen the participant(s) previously, ask if anything has changed since the last visit.

c) T-Tell them all about family planning methods

- Tell them which methods are available.
- Ask which methods interest them and what they know about the methods.
- Briefly describe each method of interest and explain how it works, its advantages and disadvantages, and possible side-effects.

d) H-Help them to choose a method

- To help them choose a method of contraception, ask them about their plans and family situation. If they are uncertain about the future, begin with the present situation.
- Ask what the spouse/partner likes and wants to use.
- Ask if there is anything they cannot understand, and repeat information when necessary.
- When the chosen method is not safe for them, explain clearly why the method may not be appropriate and help them choose another method.
- Check whether they have made a clear decision and specifically ask, 'What method have you decided to use?'

e) **E-Explain how to use a method**

After a method has been chosen:

- Give supplies if appropriate.
- If the method cannot be given immediately, explain how, when and where it will be provided.
- For methods like voluntary sterilization the participant will have to sign a consent form. The form says that they want the method, have been informed about it, and understand this information. You must help the individual understand the consent form.
- Explain how to use the method.
- Ask the participant to repeat the instructions.
- Describe any possible side-effects and warning signs, and tell them what to do if they occur.
- Ask them to repeat this information back to you.
- Give them printed material about the method to take home if it is available.
- Tell them when to come back for a follow-up visit and to come back sooner if they wish, or if side-effects or warning signs occur.

f) **R-Appoint a return visit for follow-up**

At the follow-up visit:

- Ask the participant if she is, or they are, still using the method, and whether there have been any problems.
- Ask if there have been any side-effects.
- Reassure the participant/s concerning minor side-effects. Explain that the side effects are not dangerous and suggest what can be done to relieve them.
- Refer for treatment in the case of severe side-effects.
- Ask the participant if they have any questions.

If the participants want to use a different method, tell them about other methods and help in this choice. It is important to remember that changing methods is not bad. The main thing is that they can choose a method which is acceptable and appropriate. If the participants now want to have a child, help them to discontinue the use of their current method of family planning. Make sure the participants know when and where to go for prenatal care when the spouse becomes pregnant.

8. *Very important points to note while counselling a voucher participant*

Respecting the rights of the participant is essential to the quality and continuity of family planning services, including counselling. Below, are the principles of the participant's rights in all aspects of family planning services.

Every participant has the right to:

1. **Information** — to learn about their reproductive health, contraception and abortion options.
2. **Access** — to obtain services regardless of religion, ethnicity, age, and marital or economic status.
3. **Choice** — to decide freely whether to use contraception and, if so, which method.
4. **Safety** — to have a safe abortion and to practice safe, effective contraception.
5. **Privacy** — to have a private environment during counselling and services.
6. **Confidentiality** — to be assured that any personal information will remain confidential.
7. **Dignity** — to be treated with courtesy, consideration and attentiveness.
8. **Comfort** — to feel comfortable when receiving services.
9. **Continuity** — to receive follow-up care and contraceptive services and supplies for as long as needed.
10. **Opinion** — to express views on the services offered.

(Adapted from Huezo and Diaz, 1993; some excerpts adapted from www.open.edu/openlearncreate/mod/oucontent/view.php?id=138&printable=1)
