# Supplementary material for: Provision of family planning vouchers and early initiation of postpartum contraceptive use among women living with HIV in southwestern Uganda: A randomized controlled trial
Source: PLoS Med. 2019 Jun 21;16(6):e1002832. doi: 10.1371/journal.pmed.1002832 (PMC6588214; doi:10.1371/journal.pmed.1002832)
Supplement: S2 Text — (PDF) [file pmed.1002832.s002.pdf]

FOR OFFICE USE ONLY	Date of submission	Date considered	Approval granted?
Application No. *(Yr/No)* 10/08-16	15/08/2016	[illegible]/08/2016	~~yes~~ / no
Signatures			(Chair)

THIS FORM MUST BE TYPEWRITTEN

MBARARA UNIVERSITY OF SCIENCE AND TECHNOLOGY

APPLICATION FORM FOR MUST INSTITUTIONAL REVIEW COMMITTEE APPROVAL

ALL QUESTIONS MUST BE ANSWERED. ANY FORM STATING "SEE PROTOCOL" WILL BE RETURNED. (This form must stand complete in itself).

PLEASE PROVIDE COPIES OF THIS FORM AND THE ORIGINAL PROPOSAL AS STATED IN THE GUIDELINE

AS FAR AS POSSIBLE YOU SHOULD RESTRICT ALL ENTRIES TO THE SPACE PROVIDED ON THIS FORM
Please use a typing font that is easily distinguishable from the questions of the form
NB This form is available on diskette from the Research Support Office

NAME OF APPLICANT: Esther Atukunda Cathyln, Mbarara University of Science and Technology

Have you submitted this proposal to the Mbarara University, Institutional Ethical Review Committee before?

No	Yes	Date and outcome:
**NO**		

If you are re-submitting a proposal, please emphasize how the proposal has been amended in the light of previous recommendations from the MUST, Faculty of Medicine Research & Ethics Committee or Institutional Committee

NOT APPLICABLE

If this proposal is for work that will go towards a higher degree (e.g. M.Med or PhD), please state name and Department of Supervisor(s):

1. **Professor Celestino Obua, MD, PhD. VC MUST/ Department Pharmacology and Therapeutics**
2. **Professor Amon G Agaba, MD, PhD. Assoc Dean, FOM. MUST**
3. **Dr. Lynn T. Matthews, MD, MPH. Assistant Professor, Harvard Medical School; Massachussetts General Hospital, Global Health-Division of Infectious Disease**

SECTION A

STUDY OUTLINE

A.1 TITLE OF Study: The impact of a family planning support intervention on pregnancy desires and contraceptive use among recently postpartum HIV positive women delivering at MRRH

A.2 SUMMARY

Explain why this study is being conducted, using lay terminology.

Guidance note:

Please convey what you think is the importance of the research and WHY it is being carried out.

HIV status and availability of ART have been found to widely influence the desirability and expectations to have children among childbearing women living with HIV (Nieves et al., 2015; Kaida et al., 2011). Up to 85% of the pregnancies within 3 years following ART are often unwanted, often leading to illegal abortions and maternal deaths (Desgrees-Du-Lou et al., 2002).

Uganda's HIV epidemic has declined over time to about a sero prevalence of 7.2% and this decline has been attributed in part to improved prevention behavior like condom use, reduction in multiple concurrent partnerships and other strategies to prevent unwanted pregnancies. Specifically, HIV prevalence is estimated at 7% among women attending antenatal clinics in Uganda (UN, 2010). Whereas ART availability has improved general health outcomes through viral load suppression and immune reconstitution, there is still a high unmet need for family planning among HIV positive women in Uganda (Snow et al., 2011), where modern contraceptive prevalence is at 33% in the general population despite the widespread promotional messages across the country. This continues to expose HIV positive women to increased risks of unwanted/unplanned pregnancies, perinatal HIV transmission, pregnancy complications, and increased economic burden of care for self and others among others.

The high average fertility rate of 5.9 children per woman in Uganda may further complicate the immunity and wellbeing of the already burdened HIV positive women and their families even amongst those already accessing HIV care (Muyindike et al., 2012; Homsy et al., 2009).

Supporting HIV positive women to delay or prevent an unwanted pregnancy may improve women's health through family planning choices, regular health reviews, and support. A cohort study that was done in Mbarara amongst HIV-infected women documented 50% of the enrolled women having no desire for a (another) child during the 4 years since ART initiation, 51% had a serodiscordant partner, with only 45% using effective contraceptive method. Key predictors of contraceptive use were family planning goals within the DYAD. One of the key predictors of effective contraceptive use was male partner involvement and CD4 count. The WHO recommends dual contraception (use of condoms and a hormonal or permanent method) to prevent both HIV transmission and unwanted pregnancies (WHO,2014).This prospective intervention study aims at providing information on whether continuous family planning accessibility and support has a measurable impact on pregnancy intentions and contraceptive usage among postpartum HIV positive mothers delivering at MRRH, SW Uganda.

A.3 OBJECTIVES

List the major objectives/hypothesis, which have governed your choice of study design

General objective

To test the effect of a family planning availability and support intervention on pregnancy intentions and use of effective contraception among recently postpartum women living with HIV who delivered at MRRH.

Specific objectives

1. To assess the influence of the intervention on pregnancy intentions among recently postpartum HIV positive mothers delivering at MRRH, SW Uganda one year after the intervention.

INSTITUTIONAL ETHICAL REVIEW COMMITTEE
MBARARA UNIVERSITY OF SCIENCE AND TECHNOLOGY
SUCCEED WE MUST
P. O. BOX 1410, MBARARA

2. To assess the influences of the intervention on contraceptive uptake among recently postpartum HIV positive mothers delivering at MRRH, SW Uganda
3. To identify factors that influence pregnancy intentions and contraceptive use
4. To assess the influence of the intervention on the incidence of unintended pregnancy among recently postpartum HIV positive mothers delivering at MRRH, SW Uganda
5. To assess the influence of the intervention on the well being of recently postpartum HIV positive mothers delivering at MRRH, SW Uganda
6. To explore decision making challenges and perceptions of family planning among HIV positive women.
7. To assess the role of sexual partners on pregnancy intentions and effective contraceptive use

A.4 METHODOLOGY

Outline how you intend to achieve the objectives of the study.

Guidance notes:

For each objective/hypothesis:-

- *define the target population*
- *describe how the sample(s) is(are) to be recruited from the target population(s)*

Even if the main thrust of the research is biomedical, the rationale behind your use of social science methods (e.g. patient interviews) should be clear.

describe how the sample(s) is(are) to be recruited from the target population(s)

This will be a prospective randomized controlled trial of 2 arms.

I. We intend to enroll a total of 320 HIV positive women delivering at MRRH. These will be equally randomized into the intervention arm (Family planning support) and control group (no family planning support, except the routine counseling following discharge). These women will be followed for 1 year. Eligibility will also require access to a mobile phone.

I. **Intervention group**. Following delivery, they will be given a voucher to freely access any of the five family planning options of their choice. A voucher will also be given to the spouse/sexual partner due to its identified effect on family planning utilization (Ashraf et al., 2014). Although family planning is already free, there are usually stock outs. The voucher will therefore be an incentive to easily access the family planning timely (maximum of 1 hour waiting time) and continuously from MRRH. The voucher will have a 3 months expiry date following discharge. These include; condoms (40 condoms), injectables, contraceptive pills, copper IUD and contraceptive implants. The women will be contacted at 6 months post-partum for initiation of family planning by a well-trained study research assistant. The choice of family planning will be entirely up to the participants. After 6 months postpartum, those women who select oral contraceptive pills, women will be sent daily [adherence support] reminders for the first 4 months, then weekly reminders (via SMS) for the next 4 months. This level of SMS support has been found to have a positive impact on adherence (Haberer et al., 2016). Sexual partners/regular spouses of women in intervention arm will also receive these reminders weekly [but not daily or monthly]. The reminders will also be sent monthly if one chooses an injectable contraception [consider whether it's the 2-month or 3-month injectable]. Daily reminders will also be sent for women who choose male or female condoms. Routine reviews on family planning will be done for all women alongside their routine visits at the HIV clinic or post-natal PMTCT visits. Interviews with study participants will be done at baseline, 6 and 12 months postpartum. The spouses/ sexual partners will be contacted, enrolled and interviewed at baseline, 6 months and at 1 year.

Control group: These will be counseled on family planning at discharge and enrolled. They will be interviewed again at baseline, 6 months and 1 year. Data on reproductive history, HIV/AIDS experience, socio-sexual relationships, fertility aspirations, use and knowledge of family planning, decision making, and perceptions on Family planning, alcohol use, food security, education, socio-economic, side effects or undesirable effects, economic and social support will be documented.

INSTITUTIONAL ETHICAL REVIEW COMMITTEE
MBARARA UNIVERSITY OF SCIENCE AND TECHNOLOGY
SUCCEED WE MUST
P. O. BOX 1410, MBARARA

Natural family planning methods like lactation amenorrhea, withdrawal methods and others have been presented with mixed literature, painting a grim picture on their effectiveness as standalone contraceptive methods especially among HIV positive women. All women are advised to exclusively breast feed for 6 months while on ART (MoH, 2015). Because hormonal contraception inclusive of estrogen interferes with breast milk production among lactating mothers, progestin-only methods will be recommended to the women.

I. Randomization:

Consecutive numbers ranging from 001 to 320 will be computer generated to indicate the 2 main groups into which mothers can be randomly assigned and enrolled. These consecutive numbers will be labeled on top of the opaque envelopes. To be eligible, mothers must be HIV positive, have had a delivery within the last 48 hours, above 18 years of age, anticipating to begin family planning post partum, have a spouse/sexual partner whom we may contact or invite to participate in the study, must be mentally stable and qualify for any family planning method available. Once eligibility of a mother is confirmed, they will be invited to participate in the study.

The aim of the study and details of the procedures to be involved in the trial, potential side effects and therapeutic benefits will be explained before randomization occurs. Once the mothers consent to participate in the study, a study number will be allocated by the nurse research assistant (**who will be recruited to work on the ward/post natal clinic for this study**) by taking the next in a series of similar opaque envelopes provided to conceal allocation of groups. These opaque envelopes will be labeled with computer-generated list of numbers with group allocation (either Intervention group or Control Group) of the mothers randomized in blocks of 20. Nurse research assistants will be blinded to the group allocation until eligibility and study participation is confirmed. They will also be blinded to hypothesis of the study.
A blood sample will be drawn at baseline to confirm the HIV status and or do a CD4 cell count in case it is unknown at the time of enrollment. This will be done to support women enroll for the recommended Option B+ incase the women are not yet lifelong ART].

A different study nurse will be enrolled and trained to specifically collect data from participants. The socio-demographic data and obstetric characteristics will be documented. Other documented potential covariates of contraceptive use like participant age, primary partner's age, primary partner's HIV status, personal and partner fertility desires, number of children, most recent CD4 cell count, Efavirenz-containing ART Regimen (due to its association in decreasing levels of hormonal contraception thus recommending dual methods and its teratogenicity concerns-although these concerns have been found to be exaggerated), socio-economic status, body mass index, education level, time on ART . Prior contraceptive use experiences, fertility desire/ pregnancy intentions or aspiration will be measured using the CDC pregnancy Risk Assessment Monitoring System Instrument (Ahluwalia et al., 1999)

Effective contraception use will be defined as consistent use [both self report and observational chart review from study nurse at the family planning clinic] of a family planning method (including consistent condom use) , including dual contraception (use of condoms and another contraceptive method-hormonal or permanent) for HIV-sero discordant couples/partners (for participants who know partner's status). A primary partner will be defined either as a regular spouse, who is also a regular sexual partner or the most recent sexual partner if no main partner is named. These sexual partners will also be enrolled and interviewed at baseline and at 1 year. In case a consented mother ends up with a serious adverse event, the time and indication will be documented and reported. In case a woman changes the contraceptive method, the reasons for the change will also be documented.

A.5 PARTICIPANTS

Please provide the following information on the participants with/from whom you expect to be collecting data:

A.5.1 Age / Sex: (please enter the expected number in each of the boxes)

	Neonates (<28 days)	Infants (1-11 months)	Young children (1-9 years)	Adolescents (10-17 years)	Adults (18 yrs & above)
Males					Up to 160
Female					320

Guidance notes:-
This age/sex breakdown helps convey how vulnerable the participants will be
If you are unable to give precise figures, state estimates and give an explanatory sentence in the space below

INSTITUTIONAL ETHICAL REVIEW COMMITTEE MBARARA UNIVERSITY OF SCIENCE AND TECHNOLOGY SUCCEED WE MUST P. O. BOX 1410, MBARARA

A.5.2 What specific measures are in place to take into account women of childbearing age?

Guidance notes:

women may have different responses to disease processes

The developing foetus may be particularly vulnerable in intervention trials

The study involves administration of accredited contraceptive (hormonal or IUD) 6weeks after the delivery of the baby. The contraceptives are not known to circulate into the lactating milk. These are; condoms, injectables, contraceptive pills, IUDs and implants. However, estrogen-containing contraceptives have been found to decrease milk production and therefore progesterone only methods or non-hormonal methods are recommended for lactating mothers.

A.5.3 Describe how and where the participants are to be recruited?

Guidance notes:

This is distinct from the statistical sampling method described in A.4. You should outline the procedures for recruitment of each group of participants, include details on:

- *the setting (e.g. Country, Town, District, on the ward, out-patient department ,in the home)*
- *inclusion and exclusion criteria for selection, if relevant (e.g. "Women of child-bearing age will be excluded")*

To be eligible, mothers must be HIV positive, have had a delivery within the last 48 hours, above 18 years of age, anticipating to begin family planning post partum, have a spouse/sexual partner, must be mentally stable and qualify for any family planning methods available.

The Randomized Controlled Trial (RCT) study will purposively be carried out in the Maternity ward of Mbarara Regional Referral Hospital (MRRH), south western Uganda which also doubles as a Mbarara University Teaching Hospital for the faculty of medicine. The hospital is located in Kamukuzi Division, Mbarara Municipality along 30° 20E' and 31°20'E longitude and 1°30'S and 0°30'N Latitude (DISH, 2011http://www.ugandadish.org/). The regional referral hospital receives mothers from different tribal and socio-demographic locations in Uganda. The hospital is equipped with trained staff, midwives, and obstetricians able to offer comprehensive family planning facilities. The Hospital also have a good theatre/ mini theatres for additional therapeutic/ surgical procedures that may be required for these post partum mothers. Mothers received in this hospital may also represent different mothers from various social and demographic backgrounds. MRRH records document > 12,000 deliveries annually and a 13% HIV prevalence for women delivering at the maternity ward of MRRH.

A.5.4 Please justify your choice of sample size (as described in A.4)

Pregnancy desires tend to be lower among HIV positive women (Kaida et al., 2015). Post partum pregnancy desires also tend to be low amongst women. Additionally, the "serious problem" highlighted by one third of the HIV positive pregnant women in a Ugandan study reflected an unmet need for contraception services, family planning support and unclear conjugal fertility aspirations (Snow et al., 2013).

In a superiority trial, one's null hypothesis is that the two groups are equal and alternative hypothesis (or your main hypothesis) would be that one intervention is better (superior) than the other, in this case family planning support and availability. Two primary outcomes have therefore been suggested for this study; 1) Confirmed consistent use of an effective contraceptive method at 12 months and 2), pregnancy desire/aspirations among the HIV positive women. According to Nieves et al (2015), the rate of effective contraceptive use was found to be 45% amongst HIV positive women accessing HIV care at MRRH, although most of the contraceptive use was driven by condom use. Other studies have revealed that contraceptive prevalence would be 16 to 23 percent greater if all women lived in a cluster with the highest quality of care compared with the lowest one (Mensch et al., 1996). Another study also documented that the provision of the family planning voucher had a significant impact on contraceptive experimentation and long-term contraceptive use by an increase of 18 percentage points (Ashraf et al., 2014). We therefore hypothesize that improved family planning support and voucher will increase in effective contraceptive use among HIV positive postpartum women to at least 63%. This will therefore require a total of 156 women per group and an overall total of 312 HIV positive post partum women to have a 90% chance of detecting a significant increase at the 5% level.

For the second primary outcome, Nieves et al (2015) documents a pregnancy desire/ aspiration rate of 33% for any HIV positive sexually active women attending ART clinic. Specifically, Snow et al (2014) also reported a lower and significant likelihood in desiring more children in future of 27.7% among married or cohabiting HIV-positive women when compared to 56.4% among HIV-negative women. Improved family planning accessibility and support offered through a voucher reduced the rate of births in the next year following the intervention to 6.8% (Ashraf et al., 2012). We therefore hypothesize a decrease in pregnancy or births in the next one year following our intervention to 6.8% with improved family planning availability and support among the recent post partum HIV-positive women. We shall therefore require a total of 80 women in each of the two groups and a total of 160 women in total to have a 95% chance of detecting a significant increase in pregnancy aspirations at the 5% level.

INSTITUTIONAL ETHICS REVIEW COMMITTEE
MBARARA UNIVERSITY OF SCIENCE AND TECHNOLOGY
SUCCEED WE MUST
P. O. BOX [illegible] MBARARA

The participant enrolment and analyses will be done consecutively for the two primary outcomes. Up to 160 spouses/sexual partners will also be required to match their spouses in the intervention group._Initiation of a modern family planning method at any facility of choice, rate of pregnancy, time period of family planning discontinuation and or change will also be documented.

A.6 PROCEDURES

A.6.1 What procedures or methods will be employed in the collection of data (e.g. patient interviews / focus group discussions / blood sampling / biopsies) and by whom (e.g. experienced facilitator / social scientist / teacher/ qualified doctor / nurse, auxiliary, etc.)?

Attach additional sheets if necessary.

Method	To be carried out by:
Patient interviews	Nurse Research assistants
Observations	Nurse Research assistants
Data entry	Qualified data clerk

A.6.2 State the extent to which the procedures to be used are a part of usual clinical management (if appropriate).

- Family planning methods and proposed observations are routinely done to at post natal clinics 6 weeks following child birth.

A.6.3 Please indicate that the persons identified in A.6.1 are competent to carry out these procedures. List any training of staff that may be required prior to commencement of the study.

The project personnel will include highly qualified midwives/nurses to administer family planning methods, collect a blood sample for HIV testing/CD4 cell count and collect data. Highly specialised and qualified doctors at the ISS clinic will also review these mothers. A pharmacy technician will prepare the proposed treatments and a data clerk will double check and enter data.

A.7 ANALYSIS

A.7.1 What are the major statistical (or other) methods that you intend to use to analyse the data to fulfil each of the objectives/hypothesis stated in A.3

Data Preparation

All data will be cross checked for completeness before entry. Data will be coded and entered independently into the excel sheet and exported into STATA Version 12 for statistical analysis. For each variable, a reference category will be identified.

3.4.2 Data Analysis Plan

Data analysis will be by intention-to-treat (ITT) consisting of all mothers randomized and thus supposed to be treated (Lesaffre, 2008). Descriptive statistics will be used to describe key characteristics of study participants. Different variables will be explored for normal distribution. Selected demographic, clinical, post-natal factors will be compared for differences and similarities in the groups. We will compare dichotomous outcomes between study groups by estimating crude odds ratios with 95% confidence intervals, and testing for differences between groups with chi-squared testing and a level of significance of 0.05. Continuous outcomes will be compared using t-tests. A Mantel-Haenszel test will also be done to control for different variables. Crude relative risks will also be derived. Relative risks with 95% confidence Interval (two tailed) will be calculated and used to compare contraception effectiveness and pregnancy intentions in all groups. Factors associated with effective contraception use and pregnancy intentions will be explored using univariate analysis and for results whose p value ≤ 0.10, a multivariate analysis will be done to rule out confounding factors.

3.4.3 Data Reporting

The means will be presented with their standard deviations. Relative risks and odds ratios will be presented with their 95% confidence Intervals. Two-tailed statistical analysis will be used and statistical significance will be defined at level of $p < 0.05$.

INSTITUTIONAL ETHICAL REVIEW COMMITTEE
MBARARA UNIVERSITY OF SCIENCE AND TECHNOLOGY
SUCCEED WE MUST
P. O. BOX 1410, MBARARA

A.8 QUALITY ASSURANCE

A.8.1 What procedures are in place to ensure the quality of the data?
Guidance notes:
For qualitative data (for example) what procedures will be used to check translations or compare data obtained from different sources?
For quantitative data (for example) how will transcription errors be minimised?
Give some detail on how methods are going to be piloted, if appropriate

- An instrument to collect the required data has been adapted from the tools used by other researchers in other sites.
- Computer-generated numbers will be used to randomize mothers into this study. These numbers will be in opaque envelopes to blind the observers on the group to which the next mother will be randomized and observed. The Investigator or researchers/ observers will not have prior knowledge of the treatment allocated to the next patient to avoid bias into the random allocation process.
- Details of the aim and procedures to be involved in the trial for the three groups, potential side effects and therapeutic benefits will be explained to the eligible patients on admission by the nurse research assistant before randomization occurs. Once the mothers consent to participate in the study, opaque envelopes containing computer-generated consecutive numbers from 001-320 will then be opened by the researcher and the random group allocation of the mothers will be communicated.
- Improved routines at the time of discharge will be observed for all mothers on the ward regardless of the study intervention. Family planning counseling and advice will be availed to all mothers at discharge.
- Routine reviews, counseling and support will be done to ensure adequate management of side effects and other family planning concerns that may arise during the course of the study.
- Internationally recognized brands of contraceptives or their equivalent from national Medical stress (NMS) will be used for this study
-

- Two Nurse research assistants will be **recruited** and **trained** as observers and data collectors to increase accuracy and consistency in documenting the needed data. They will be assisted by the post natal clinic staff and these research assistants will be blinded to the hypotheses of this study. To minimize observer bias and facilitate participant randomization, one nurse research assistant will deal with enrollment, family planning counseling and support while another will entirely follow up participants and collect the required data at different time points. Senior Obstetricians will supervise the trial and administration of contraceptives on post natal clinic.
- Mothers will be free to withdraw from one family planning method to another following their doctors advice.

A.9 DISSEMINATION OF RESULTS

Please outline what plans you have for dissemination of results.

Guidance notes:

Where possible a mechanism should be in place to inform study participants of the outcomes of the study.

It is important that study findings are made known to local services / policy makers before they are discussed (e.g.) at international scientific meetings

Particularly, the dissemination of results for this study is motivated by both the academic and social impact, with a focus on the practical benefits for the key stakeholders. The following points are highlights of the approach:

- Presentation of the project on the MUST website [if possible] www.must.ac.ug/research
- Local meetings with key stakeholders in health policy, pharmaceutical sector, and other sectors of civil society and government
- Presentations at institutional, national and international conferences
- Specially tailored open-source materials (such as policy briefs, articles for magazines that reach policymakers or presentations to policymakers)
- Dissemination of research findings through information networks operating at various levels
- Submission and publication of research findings to international peer-reviewed journals
- Dissemination of research reports and other project outputs on an open section of the website.

INSTITUTIONAL ETHICAL REVIEW COMMITTEE
MBARARA UNIVERSITY OF SCIENCE AND TECHNOLOGY
SUCCEED WE MUST
P. O. BOX 1410, MBARARA

SECTION B

CONSEQUENCES FOR THE LOCAL COMMUNITY / ENVIRONMENT AND PARTICIPANTS

B.1 Outline the potential adverse effects, discomfort or risks that may result from the study in the following areas:

B.1.1 Participants

Guidance note:

In addition to the physical effects of tissue sampling (for example blood sampling) it should be borne in mind that interviews and focus group discussions may sometimes trigger painful or distressing memories (e.g. questions about sexual practice or the death of a child)

1. There are some questions that will be asked and have been classified 'sensitive or personal'. In case the participants feel some anxiety answering some of them, they may choose not to answer some questions they feel uncomfortable

2. There is the risk of discomfort and or pain when a contraceptive method is being administered or a blood sample for HIV testing and CD4 cell count is being drawn. There is also a small risk of bruising, scarring or infection. However, care will be taken to professionally administer these routinely used methods with good infection prevention techniques. A participant may also refuse a particular contraceptive method and chose another that makes her comfortable at any time.
3. There is a risk of side effects following administration of these contraceptive methods, like longer periods, dysmenorrheal, acne, weight gain, etc. In case this happens, routine procedures will be followed to give the participant additional family planning support, counseling and advice, therapeutic medications or procedures to change, treat or prevent further occurrences. Trained health professionals will also be available during the study to provide professional advice and answer any further questions

B.1.2 Investigators

Guidance notes:
Include here (for example)

- *the biomedical risks to investigators (including local staff) involved in tissue sampling (e.g. Hepatitis B, HIV)*
- *the psychological consequences for social science investigators exposed to narratives of violence or severe grief*
- *the risks from the environment (e.g. in a war zone)*

There are anticipated risks to Hepatitis B or HIV especially since the study is targeting administration of the family planning method among HIV positive women.

B.1.3 Members of the public

We do not expect any adverse effects, discomfort or risks to members of the public.

B.2 Outline what steps will be taken to minimise the adverse effects, discomfort or risks described above.

B.2.1 For participants

Guidance notes:
In biomedical research, appropriate use of anaesthesia prior to procedures (for example) is important.
For social science research it may be necessary to ensure that counselling services are available for those who re-live traumatic experiences through (for example) an in depth interview.

- There are some questions that have been classified 'sensitive or personal', that may cause the participants to feel some anxiety answering some of them. In this case, they may choose not to answer questions they feel uncomfortable
- The risk of discomfort, bruising, scarring or infection when contraception is administered or a blood sample is being drawn will be minimized by engaging highly trained personnel in offering family planning services.
- The risk of side effects following contraceptive administration for the intervention groups will be managed by following routine procedures to give the participant support, counseling, advice or additional therapeutic medications or procedures to treat or prevent effects. Trained health professionals will also be available during all post natal visits to answer any further questions
- The nurse research assistants will not discuss any information obtained from the participant with anyone other than the research and health care team
- Refusing to participate in this study at any time will not affect healthcare services that a participant would ideally obtain from the hospital.

INSTITUTIONAL ETHICAL REVIEW COMMITTEE
MBARARA UNIVERSITY OF SCIENCE AND TECHNOLOGY
SUCCEED WE MUST
P. O. BOX 1410, MBARARA

B.2.2 For investigators

Guidance notes:

Where the research may involve adverse experiences for investigators (see B.3.2), de-briefing / support meetings may be important

Protective gear like aprons and gloves will be readily provided and availed for clinic staff at all times to prevent any risk of HIV transmission during administration of contraceptives and drawing a blood sample

Procedures to obtain post exposure prophylaxis will also be streamlined and services availed to all staff at all time through the routine hospital policy.

Training on infection control will be done and emphasized for all the staff involved in this study.

B.2.3 For members of the public

ADVERSE EXPERIENCES ARE NOT EXPECTED

B.3 CONSEQUENCES FOR LOCAL HEALTH SERVICES

B.3.1 What demands will this research place on local health services?

Guidance notes:

For example, how much of a nurse's usual work time will be taken up in acting as an interpreter for an outside investigator?

During the study, which is expected to last a period of about 2 years, the nurse RAs may require to use the routine office space at the maternity ward, post-natal ward and post-natal clinic to contact, randomize, enroll and or document observations. They may also take up some available working space and use the available shelves to keep contraceptives. The nurse RAs may also require assistance from fellow nurses/midwives in administering contraceptive methods or taking required observations. Nurse research assistants speaking the local language will however be recruited for this study

B.3.2 Detail how the design of the research project takes into account the demands described in 3.1.

Guidance notes:

Disruption to routine services should be kept to a minimum.

The study will not disrupt the routine services. All contraceptive methods to be used in this study are routinely used and recommended by the MoH. NMS also routinely supplies them to health centers. These include; condoms, injectables, contraceptive pills, IUDs and hormonal implants.

B.4 CONFIDENTIALITY AND PRIVACY

B.4.1 What steps will be taken to ensure privacy and confidentiality for participants?

1. All Research Assistants will undertake a course on Research on Human subjects before the study commences
2. Participants in this study will be ensured of their privacy. The research assistant will not discuss any information obtained from the participant anyone other than the research and health care team
3. Research data will be collected on a password controlled computer and each participant will be assigned an anonymized study identification number. This data will be entered and maintained in an electronic format on the computer that will be password protected at all times. The hard copies and consent forms will be kept in a lockable cabin for safety and privacy. Researchers will take every extra precaution to always exclude any information to identify participants personally.
4. All research records will be handled as confidentially as possible. All research records will be coded with an anonymised study ID so that no person outside the study group can identify the participant. No individual identities will be used in any reports or publications that result from this study. No individual identities will be included in all the data shared with other researchers.
5. Informed consent will always be sought from the participants before enrollment into the study

B.5 **INFORMED CONSENT**

B.5.1 Information given to participants:

Please indicate what you will tell the participants in <u>simple language</u>. The purpose of the study, type of questions that will be asked, and procedure or treatment which will be applied should be described and reference should be made to possible side effects, discomfort, complications and/or benefits. If a specific consent form is available, please attach.

1. It will be made clear to the participant that he/she is free to decline to participate or to withdraw at any time without affecting healthcare services obtain from the hospital/clinic or any other disadvantage or prejudice. The consent form has been attached.

INSTITUTIONAL ETHICAL REVIEW COMMITTEE
MBARARA UNIVERSITY OF SCIENCE AND TECHNOLOGY
SUCCEED WE MUST
P. O. BOX 1410, MBARARA

5.2 Outline who will deliver the above information and how?

The information will be delivered by nurse Research Assistants of the study.

- They will share information about the study with the participants, i.e, introduce themselves, explain the purpose and type of the research to be carried out, and invite the participants to join the study, on a voluntary basis. This will be done at the post natal ward and before discharge. This will give the participants ample time to think through the consent process and make an informed decision without coercion.
- The RAs will also explain the procedures, risks and benefits, ensure confidentiality, dissemination of results, and whom to contact later, for further questions.
- They will provide a certificate of Consent to the participants for their signatures

B.5.3 Please indicate how consent will be obtained, given local circumstances.

Guidance notes:

In some societies, the concept of giving consent on an individual basis is unfamiliar. It may be necessary to obtain consent both at community and individual level.

Obtaining consent from minors requires both consent from the guardian and, where possible, the minor.

Consent will be sought on an individual basis and only adult mothers will be approached for possible participation in the study.

B.5.4 Are any inducements to be offered to either participants or the individuals who will be recruiting them? (e.g. improved patient care / cash) (please tick appropriate box)

Yes	No
	√

B.5.5 If yes, please give details:

N/A

B.5.6 Outline any hidden constraints to consent.

Guidance notes:

Examples where hidden constraints may be important include:

- *situations where participants are employees of the investigator*
- *patients who may feel their care could be compromised if they do not consent to research initiated by their carers.*

- Patients may feel obliged or a responsibility to participate in the study fearing any sort of bias to be offered routine care in case they do not consent. However, efforts will be put to explain to the participant that participation is purely voluntary, with no disadvantages in case they chose to withdraw at whatever stage of the study. The participants will also be contacted after delivery while in post natal ward and before discharge to allow ample time to think through the consent process, the benefits and risks and consult their next of kins regarding their voluntary participation.

RESPONSIBILITY

C.1 Litigation:

In respect of any litigation which may result from this research
a) Who will provide compensation?

All family planning methods available on the market have been documented to be safe when used for contraception/birth control. Particular litigation is not envisaged in the overall research framework of this particular study. However, this issue will be brought to the attention and review of the Data and safety Monitoring committee in case safety issues do arise.

This data and safety monitoring committee will constitute the senior staff on the post natal ward to ensure safety of participating mothers. Four safety checks will be done at 25% recruitment, 50%, 75% and at the end of data collection.

(Please provide documentary evidence where appropriate.)

b) What insurance arrangements have been made by the applicant and his/her delegated assistants?

As in C.1 a. above

(Please ensure that any professional indemnity insurance is logged with the Director's office)

C.2 **DECLARATION: TO BE SIGNED BY MAIN APPLICANT**

- I confirm that the details of this proposal are a true representation of the research to be undertaken.

- I will ensure that the research does not deviate from the protocol described.

- If significant protocol amendments are required as the research progresses, I will submit these to the Mbarara University Faculty Medicine Research Ethics Committee for approval.

- Where an appropriate mechanism exists, I undertake to seek additional local Ethical Approval in the country(ies) where the research is to be carried out.

I expect the study to commence on (Date): 1st Sept, 2016 and be completed by (Date): 1st Sept 2018.

Signed [signature] Date 15/08/2016

SECTION D
APPROVALS

D.1 List research team and all collaborators.
(Please include all overseas collaborators and give their affiliations, qualifications and role in the study).

INSTITUTIONAL ETHICAL REVIEW COMMITTEE * P.O. BOX 1410, MBARARA * MBARARA UNIVERSITY OF SCIENCE AND TECHNOLOGY SUCCEED WE MUST
