# Supplementary material for: Provision of family planning vouchers and early initiation of postpartum contraceptive use among women living with HIV in southwestern Uganda: A randomized controlled trial
Source: PLoS Med. 2019 Jun 21;16(6):e1002832. doi: 10.1371/journal.pmed.1002832 (PMC6588214; doi:10.1371/journal.pmed.1002832)
Supplement: S3 Text — (PDF) [file pmed.1002832.s003.pdf]

REPRODUCTIVE AND CONTRACEPTION BEHAVIOUR FOLLOW-UP TOOL FOR PARTICIPANTS (6 MONTHS)

1. Are you breast feeding your baby?
   1.Yes 2. No

   a) If No, why?.................

   b) If yes, where did you access information about breast feeding?

---

2. In the last 6 months have you had sex? (by sex, we mean, vaginal)
   1. Yes
   2. No

Knowledge on HIV

Considering the new medicines available to treat HIV, please tell me how much you agree or disagree with the statements I read to you. I want to know your opinion, so please be honest. Do you strongly agree, agree, disagree, or strongly disagree with the statement?

3. A person with virus that has been turned off in the body (a very small amount of virus in the body) cannot pass the virus to someone else

   ☐**Strongly Agree** ☐**Agree** ☐**disagree** ☐**Strongly Disagree**

4. If a cure for AIDS were announced, I would stop practicing safe sex (abstinence, being faithful, or using a condom)

   ☐**Strongly Agree** ☐**Agree** ☐**disagree** ☐**Strongly Disagree**

5. People with a very small amount of virus in the body do not need to worry about infecting others with HIV

   ☐**Strongly Agree** ☐**Agree** ☐**disagree** ☐**Strongly Disagree**

6. I believe that new drug therapies make people with HIV less able to pass the virus to other people

   ☐**Strongly Agree** ☐**Agree** ☐**disagree** ☐**Strongly Disagree**

7. If my partner and I were both on HIV treatments, it would be OK to stop using condoms

   ☐**Strongly Agree** ☐**Agree** ☐**disagree** ☐**Strongly Disagree**

8. Now that HIV medicines are available, I want to have a baby within the next 2 years because I will live long enough to raise a child

☐**Strongly Agree** ☐**Agree** ☐**disagree** ☐**Strongly Disagree**

For women only:

I am not worried about becoming pregnant because of available HIV treatments ☐ **Strongly Agree** ☐**Agree** ☐**disagree** ☐**Strongly Disagree**

9. It would be OK to stop HIV medicines if they made me sick, especially if I were pregnant

☐**Strongly Agree** ☐**Agree** ☐**disagree** ☐**Strongly Disagree**

As you may know, a person may get the AIDS virus through sexual activity. To help prevent the spread of AIDS, we need to know more about all the different types of sexual practices that people have. Some of these questions need to be rather detailed and personal. Since this survey is confidential, no one will know your answers. We would appreciate your participation in answering these questions as openly as possible.

10. In your opinion, which one would you be worried about if you had unprotected sex?

1. Pregnancy 2. HIV transmission 3. Other (specify)......

11. Why? (please explain)..

12. Are you currently taking ARVs to manage your HIV?
    1. Yes
    2. **No**

13. When did you start using ARVs? ***MM*** _____ ***YY*** ________

14. How would you rate your health before starting ARV?
    1. Poor
    2. Fair
    3. Good
    4. Very Good
    5. Excellent

15. How would you rate your health now?
    1. Poor
    2. Fair
    3. Good

4. Very Good
5. Excellent

16. Have you had any other serious condition or illness in the last 6 months (specify)

1. Yes (specify) .... 2. No

17. Were you admitted in hospital/ clinic/ or see a doctor for this condition

1. Yes 2. No

Intimate Partner Violence

People in relationships often disagree and sometimes these fights become physical. I am going to ask some questions about this. I want you to speak freely and remember that everything you say will be confidential.

18. . Have any of your sexual partners **ever** done any of the following? **Read all options before pausing**. **Pushed, pulled, slapped, or held you down? Punched you? Kicked you or dragged you? Tried to strangle or burn you? Threatened or attacked you with a gun/knife/other weapon?**

☐ Yes ☐ No ☐ Don't know ☐ Refused

19. Have any of your sexual partners done any of the following? Read all options before pausing. Pushed, pulled, slapped, or held you down? Punched you? Kicked you or dragged you? Tried to strangle or burn you? Threatened or attacked you with a gun/knife/other weapon?

☐ Yes ☐ No ☐ Don't know ☐ Refused

20. Have **you ever** physically hurt or threatened a sexual partner, including: **Read all options before pausing**. Pushed, pulled, slapped, or held him down? Punched him? Kicked or dragged him? Tried to strangle or burn him? Threatened or attacked him with a gun/knife/other weapon?

☐ Yes ☐ No ☐ Don't know ☐ Refused

21. Have you physically hurt or threatened a sexual partner, including: **Read all options before pausing**. Pushed, pulled, slapped, or held him down? Punched him? Kicked or dragged him? Tried to strangle or burn him? Threatened or attacked him with a

gun/knife/other weapon?

☐ Yes ☐ No ☐ Don't know ☐ Refused

22. Has anyone **ever** physically forced you to have sex with him when you did not want to?

☐ Yes ☐ No ☐ Don't know ☐ Refused

23. Has anyone physically forced you to have sex with him when you did not want to?

☐ Yes ☐ No ☐ Don't know ☐ Refused

24. Have **you ever** physically forced anyone to have sex with you when he did not want to?

☐ Yes ☐ No ☐ Don't know ☐ Refused

25. Have you physically forced anyone to have sex with you when he did not want to?

☐ Yes ☐ No ☐ Don't know ☐ Refused

ATTITUDES/ PERCEPTION ABOUT FAMILY PLANNING /CONTRACEPTION

Now I'd like to ask you some questions about contraception.

26. Have you or your sexual partner initiated/started on any family planning method in the last 6 months?

1. Yes 2. No

(READ THESE ALOUD, ONE BY ONE)

27. Has any of your sexual partners undergone sterilization? In other words, he had an operation (vasectomy) to avoid having any(more) What year? Don't Know year,

☐ Yes ☐ No ☐ Don't know ☐ Refused

28. To the best of your knowledge, have you or any of your partners used anything else to prevent pregnancy **in the past 6 months) [**This may include using hormonal contraceptive methods, condoms, or behavioural methods].

☐ Yes ☐ No ☐ Don't know ☐ Refused

In the past 6 months; did you,

29. Take a pill every day to avoid becoming pregnant? 1.Yes 2. No

(These are known as "the pill" or "oral contraceptives").

30. Get an injection from the clinic every few months to avoid pregnancy? These are known as "injectables". 1.Yes 2. No

31. Ever taken pills after sex to avoid pregnancy? (This is also known as "the morning after pill" or "emergency contraception").

☐ Yes ☐ No

32. Have a loop or coil inside you that was placed there by a doctor or nurse to keep from becoming pregnant? (This is known as an "IUD")? When?........ ….Don't Know ……..*Please check to confirm dates*

☐ Yes ☐ No

33. Have anything placed inside your skin like arm, leg, etc by a doctor or nurse to keep from becoming pregnant? (This is we mean contraceptive "IMPLANTS")? What date?........ Don't Know ……….. *Please check to confirm dates*

☐ Yes ☐ No

34. Did any of your partners ever use condoms, a rubber sheath placed on the penis before sexual intercourse, to prevent pregnancy and/or prevent HIV transmission?

Ask about each of the sexual partners

☐ Yes ☐ No

35. How frequently did you use a condom when having sex **in the past 6 months**,

- Rarely (once in a while)
- Sometimes (about half of the time)
- Frequently (more than half of the time)
- Always
- Don't Know
- Refused

36. Ever pull out before climax/ejaculation to prevent pregnancy? This is known as "withdrawal". ☐ Yes ☐ No ☐ Dont know ☐ Refused

37. Did you and this partner ever avoid sexual intercourse on days of the month when you are most likely to get pregnant? This is also known as the"rhythm method" or "safe period". ". ☐ Yes ☐ No ☐ Dont know ☐ Refused

38. Did you or your partners use any other method to prevent pregnancy?

☐ Yes ☐ No ☐ Dont know ☐ Refused

39. Please specify..

40. Which of the following methods of family planning have you ***<u>used/ heard about in the last 6 months</u>***? **[READ OUT RESPONSES]**

Device	HEARD ABOUT	USED	When initiated (in weeks postpartum) (self-report)	When initiated (in weeks) (record/audit)	Discontinued/ switched from
1. Condoms					
2. Oral Pills					
3. Foaming Tablets					
4. Injection					
5. Intrauterine Device					
6. Implants					
7. Rhythm Method/Moonbeads					
8. Lactation Amenorrhea					
9. Withdrawal					
10. Vasectomy					
11. Tubal Ligation or Hysterectomy					
12. Traditional Medicines/Herbs					

41. How was your experience while on this pregnancy control. Please explain in detail.

42. Was your last pregnancy planned? ". ☐ Yes ☐ No

43. Please explain what happened...........

44. Does your partner plan to have a child in the future?

a. Yes, he would like to have another child/children
b. No more/none

c. Undecided/unsure

45. Have you, your baby, parent, sibling or spouse been admitted in hospital in the last 6 months? 1. Yes (specify)………………… 2. No

46. While in hospital, were u able to continue on your Family planning method, ART or other medications?

1. Yes 2. No (explain why)

....

47. Are you on any herbal medicine? (specify, and for what?)………………

48. On a scale of 1-10, how effective do you think the following family planning is effective in preventing unwanted pregnancies?

Device	scale	REASONS	WOULD RECOMMEND IT
1. Condoms			
2. Oral Pills			
3. Foaming Tablets			
4. Injection			
5. Intrauterine Device			
6. Implants			
7. Rhythm Method/Moonbeads			
8. Lactation Amenorrhea			
9. Withdrawal			
10. Vasectomy			
11. Tubal Ligation or Hysterectomy			
12. Breast feeding			
13. Traditional Medicines/Herbs			

49. What method(s) are you ***<u>currently using</u>***?/ why??

Device	Yes	Self-report	Record audit	Specify date initiated	Never	REASONS FOR YOUR CHOICE
14. Condoms						
15. Oral Pills						
16. Foaming Tablets						
17. Injection						
18. Intrauterine Device						
19. Implants						
20. Rhythm Method/Moonbeads						
21. Lactation Amenorrhea						
22. Withdrawal						
23. Vasectomy						
24. Tubal Ligation or Hysterectomy						

25. Traditional Medicines/Herbs						

50. Have you changed contraception that you were using in the last 6 months?

1. Yes 2. No

51. Which one?...

52. How long do you plan to be on this current method? .... (name it)………..

53. Explain to us in detail your reasons for your choice...........

54. Ever heard about it before? 1. Yes 2. No

55. From where?

1. Friend 2. Health worker 3. Radio/ TV 4.spouse 5.other (specify).................

56. What do you know about this contraceptive method. (explain in Detail).......................

57. If using condoms, are they for **[READ RESPONSES]**:

1. Protection
2. Family Planning
3. Both

58. Reasons for changing/switching contraception.

		Yes	No
1.	Infrequent sex or no sex		
2.	Side-effects		
3.	Lack of knowledge about methods		
4.	Lack of knowledge about source—where to get them		
5.	Health concerns		
6.	Cost		
7.	Contraceptive will interact with ARV drugs		
8.	My husband/partner is opposed to use		
9.	Religious reasons		
10.	Others (specify)		

..

59. Please tick if any of these were also reasons for changing contraception

- o Causes cancer

- Causes infertility
- Makes one grow fat
- Makes one loose appetite
- Spoils my skin
- Other (List them)

60. Have you stopped/discontinued or skipped using any contraception in the last 6 months?

    1. Yes (specify)…a) stopped/discontinued………… 2. No

    b) skipped/forgot………………(specify for how long-days)………

(Please obtain both self-report and pill counts/record audits, etc)

61. Reasons for stopping/skipping contraception.

		Yes	No	How long (days)
1.	Wants to become pregnant			
2.	I discovered I was pregnant			
3.	Death of baby/child			
4.	Infrequent sex or no sex			
5.	Menopause or sterilization			
6.	Infertility			
7.	Side-effects			
8.	Lack of knowledge about methods			
9.	Lack of knowledge about source—where to get them			
10.	Health concerns			
11.	Cost			
12.	Contraceptive will interact with ARV drugs			
13.	My husband/partner is opposed to use			
14.	Religious reasons			
15.	Travel			
16.	Forgot (specify f			
17.	Others (specify)			

..

62. How long have you used family planning without stopping? ***MM _____ weeks ______ days (please confirm with records)***

63. Please tick if any of these were also reasons for stopping contraception
    - Causes cancer
    - Causes infertility
    - Makes one grow fat
    - Makes one loose appetite
    - Spoils my skin

- Other (List them)

64. Where did you most recently obtain your last method you used for family planning? Please specify when and why for each of these sources.

Accessed	Date	Reason for choice if not obtained from MRRH	Where it was administered
1. Government Hospital (specify)			
2. Government Health Center			
3. Family Planning Clinic			
4. Outreach			
5. Government Community-based distributor			
6. Private Hospital/Clinic			
7. Pharmacy/drug shop			
8. Private doctor/nurse/midwife			
9. NGO community-based distributor			
10. Shops			
11. Faith based hospital/clinic			
12. Friends/relatives			
13. Other			

65. Was there a cost the last time you obtained this method of family planning?
    1. Yes
    2. No

66. What was the cost? ___________Ush

67. Does your current husband/partner know that you are using family planning?
    1. Yes
    2. No

68. Do you feel that you can talk to your husband/partner freely about family planning?
    1. Yes
    2. No

69. How often have you and your husband/partner talked about family planning in the past 6 months?
    1. Never
    2. Once or twice
    3. Three or four times
    4. Greater than four times

70. ***"Please indicate whether you strongly agree, agree, are neutral, disagree, or strongly agree with each of the following statements."*** **[PLEASE UTILIZE SCALE]**

	Question:	Strongly Disagree 1	Disagree 2	Neutral 3	Agree 4	Strongly Agree 5
71.	My husband/partner does not approve of using contraception.					
72.	My husband/partner doesn't approve of the use of contraceptives until we've reached his desired number of children.					
73.	I need to have as many children as my husband/partner desires.					
74.	I still want to give birth to more children.					
75.	Using contraception is against God's plan.					
76.	Contraceptive use is inconvenient.					
77.	Contraceptives are too expensive.					
78.	Using contraception is bad for your health.					
79.	My religion forbids the use of contraception.					

80. I'm now going to ask you about what problems family planning methods have caused for you. Have you had any of the following since you have been on contraception,

	yes	Specify which one	no	Rate how worried you became(scale 1-5)
Nausea				
constipation				
Prolonged menstrual periods				
Loss of appetite				
Pain (specify)				
headaches				
numbness				
Rash				

Changes in your body looks (fat deposits)				
Muscle aches (specify)				
Problems having sex, eg loss of interest/lack of satisfaction				
Feel sad, down or depressed				
Others (specify)				

81. Would you recommend this contraceptive method to anyone? 1. Yes 2./No

82. Why? (please explain)...

83. Have you ever had a discussion about contraceptive use with a health care worker (nurse/doctor/counselor)?
    1. Yes
    2. No

84. If participant has never used any family planning or contraceptives, what are the reasons they did not use it?

		Yes	No
1.	Wants to become pregnant		
2.	I discovered I was pregnant before I started contraception		
3.	Infrequent sex or no sex		
4.	Menopause or sterilization		
5.	Infertility		
6.	Side-effects		
7.	Lack of knowledge about methods		
8.	Lack of knowledge about source—where to get them		
9.	Health concerns		
10.	Cost		
11.	Contraceptive will interact with ARV drugs		
12.	My husband/partner is opposed to use		
13.	Religious reasons		
14.	Others (specify)		

85. On a scale of 1-5, how safe do you think the following are to transmitting the HIV infection?
    1. Breast feeding
    2. Condoms

END: THANK YOU FOR YOUR VALUED TIME
